# Influence of Hydroxycinnamic Acids on the Maillard Reaction of Arabinose and Galactose beyond Carbonyl-Trapping

**DOI:** 10.1021/acs.jafc.4c02959

**Published:** 2024-07-05

**Authors:** Leon Valentin Bork, Nicolas Proksch, Tobias Stobernack, Sascha Rohn, Clemens Kanzler

**Affiliations:** † Institute of Food Technology and Food Chemistry, Department of Food Chemistry and Analysis, Technische Universität Berlin, Gustav-Meyer-Allee 25, 13355 Berlin, Germany; ‡ Leibniz Institute of Vegetable and Ornamental Crops (IGZ) e. V., Plant Quality and Food Security, Theodor-Echtermeyer-Weg 1, 14979 Grossbeeren, Germany; § Department of Chemical and Product Safety, Federal Institute of Risk Assessment, Max-Dohrn-Street 8−10, 10589 Berlin, Germany

**Keywords:** Maillard reaction, nonenzymatic browning, vinylcatechol, vinylguaiacol, furfural, 5-hydroxymethylfurfural, pyrrole-2-carbaldehyde

## Abstract

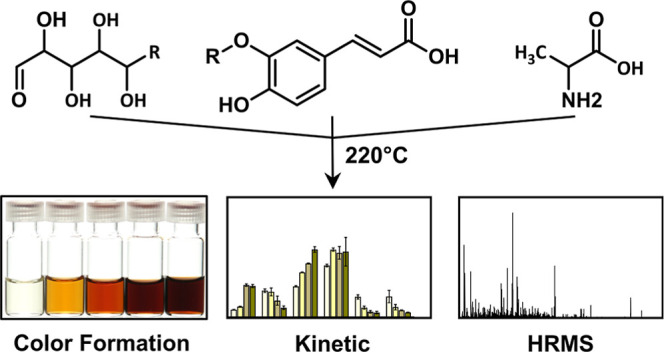

Hydroxycinnamic acids, known for their health benefits and widespread presence in plant-based food, undergo complex transformations during high-temperature processing. Recent studies revealed a high browning potential of hydroxycinnamic acids and reactive Maillard reaction intermediates, but the role of phenolic compounds in the early stage of these reactions is not unambiguously understood. Therefore, we investigated the influence of caffeic acid and ferulic acid on the nonenzymatic browning of arabinose, galactose, and/or alanine, focusing on the implications on the formation of relevant early-stage Maillard intermediates and phenol-deriving products. Contrary to previous assumptions, hydroxycinnamic acids were found to promote nonenzymatic browning instead of solely trapping reactive intermediates. This was reflected by an intense browning, which was attributed to the formation of heterogeneous phenol-containing Maillard products. Although, caffeic acid is more reactive than ferulic acid, the formation of reactive furan derivatives and of heterogeneous phenol-containing colorants was promoted in the presence of both hydroxycinnamic acids.

## Introduction

Hydroxycinnamic acids (HCA) are ubiquitous phenolic compounds in plant-based food that are frequently consumed in a western diet with an average intake of around 200 mg/day.^1^ Due to their antioxidant, anti-inflammatory, anticancerogenic, and antimicrobial properties, these compounds are considered beneficial for the human health^2,3^ and contributing to the stability of food as natural preservatives.^4^ However, their enzymatic polymerization leads to the formation of dark pigments, namely, melanins. These induce a discoloration of fresh fruits and vegetables, which is associated with lower food quality, consequently decreasing consumer acceptance.^5^ In contrast, thermally induced reactions of hydroxycinnamic acids contribute to the formation of desired aroma compounds and colorants during the processing of food rich in free and bound hydroxycinnamic acids, such as coffee,^6^ nuts,^7^ and grains.^8^

The fate of hydroxycinnamic acids during coffee roasting is of particular interest because coffee is one of the major dietary sources of brown polymers derived from nonenzymatic browning, contributing to an intake of up to 2 g per day.^9^ Although the formation of these so-called melanoidins is commonly attributed to the thermal conversion of carbohydrates and amino compounds in the complex reaction cascade of the Maillard reaction,^10^ it has been shown that coffee melanoidins consist of up to 20% of phenolic compounds.^11,12^ Apart from understanding the role of phenolic compounds in the Maillard reaction and particularly their contribution to melanoidin formation, the underlying reaction mechanisms have not been clarified so far. This knowledge is needed to understand and eventually control the nutritional, technofunctional, and even potentially toxicological effects of Maillard reaction products, especially in plant-based food.

Regarding the interaction between phenolic compounds and Maillard reaction intermediates, in particular carbonyl compounds, a concept called “carbonyl-trapping” has been established.^13,14^ The reaction mechanism underlying “carbonyl-trapping” is electrophilic aromatic substitution, where the phenolic compounds act as donors and electrophilic carbonyl compounds serve as acceptors, which has been reported for key Maillard intermediates, for example: Amadori products,^15,16^ α-dicarbonyl compounds, such as 3-deoxyosone,^14^ glyoxal,^17^ and methylglyoxal (MGO),^13^ as well as heterocyclic intermediates like 5-hydroxymethylfurfural (HMF).^18,19^ These “carbonyl-trapping” reactions are primarily discussed for flavonoids and interpreted to inhibit the Maillard reaction and thereby suppress browning by “trapping” reactive intermediates that are considered as key contributors to color formation. There are only few studies reporting such reactions for hydroxycinnamic acids,^20,21^ which are highly abundant in coffee. This is plausible as the substitution of the aromatic ring systems seems less prone to such reactions. However, recent findings showed that under roasting conditions, the adducts formed by the reaction of caffeic acid (CA) and ferulic acid (FA) with glyoxal and methylglyoxal cannot be regarded as stable and undergo subsequent oligomerization reactions, leading to intense brown colorants.^22^

As earlier investigations were focused on characterizing simple carbonyl-trapped phenol adducts and concluded that phenolic compounds inhibit the Maillard reaction, these findings have opened a novel field in nonenzymatic browning. A further in-depth investigation into this area could reveal the fundamental reactions that contribute to the formation of complex, colored, phenol-containing melanoidins.

Although the understanding of nonenzymatic browning reactions between reactive Maillard intermediates and hydroxycinnamic acids are important to comprehend the intermediary reactions that might contribute to color, it is of even greater importance to determine whether these “classic” Maillard intermediates are relevant products deriving from the heat-induced conversion of sugars and amino compounds in the presence of hydroxycinnamic acids. Therefore, the influence of the prominent hydroxycinnamic acids caffeic acid and ferulic acid on the Maillard reaction of arabinose (Ara) or galactose (Gal) with alanine (Ala) was investigated. These sugars were chosen because of their relevance for melanoidin formation in coffee: arabinogalactans, in addition to galactomannans, make up the majority of polysaccharides in green coffee beans. Additionally, arabinogalactans^23^ and arabinose^24^ were reported to be significant constituents of coffee melanoidins.^25,26^ Despite coffee beans not containing meaningful amounts of arabinose or galactose initially, (thermal) hydrolysis reactions of arabinogalactan, which are promoted under acidic conditions, as given in the presence of phenolic acids, could release the monomeric sugars.^27^ Alanine was selected as an amino acid with a comparatively inert side chain to minimize possible side reactions apart from the Maillard reaction. The reactivity of the selected compounds was characterized by incubation of the pure substances as well as of binary and ternary reaction mixtures of each substance class under roasting conditions. For evaluation, the color formation (absorbance at 420 nm), the conversion of the reactants (HPLC-UV, GC–MS), and the formation of reactive heterocyclic Maillard intermediates (HPLC-UV), more precisely furfural (FF), hydroxymethylfurfural, and pyrrole-2-carbaldehyde (PA) was monitored. The composition of potential color precursors was tentatively assigned by high-resolution mass spectrometry (HRMS). A trolox equivalent antioxidant capacity (TEAC) assay was utilized to determine the antioxidant properties of the colored reaction mixtures to derive parallels to the antioxidant properties of coffee melanoidins.

## Material and Methods

### Chemicals

l-Alanine and ferulic acid were purchased from Carl Roth GmbH + Co. KG (Karlsruhe, Germany). Acetic acid, acetonitrile, ethyl acetate, and methanol were acquired from VWR International GmbH (Darmstadt, Germany). l-arabinose, 2,2′-azinobis(3-ethylbenzothiazoline-6-sulfonate) (ABTS), caffeic acid, furfural, d-galactose, hydroxymethylfurfural, *N*,*O*-bis(trimethylsilyl)trifluoroacetamide (BSTFA) and trimethylchlorosilane, potassium peroxodisulfate, pyridine, pyrrole-2-carbaldehyde, and 6-hydroxy-2,5,6,8-tetramethylchroman-2-carboxylic acid (trolox) were purchased from Sigma-Aldrich Chemie GmbH (Steinheim, Germany). Succinic acid was obtained from Serva Electrophoresis GmbH (Heidelberg, Germany). Potassium dihydrogen phosphate and potassium hydrogen phosphate were acquired from Merck KGaA (Darmstadt, Germany).

### Incubations of Hydroxycinnamic Acids with Carbohydrates and/or Alanine

The reaction systems were prepared using 0.05 mmol of each reactant. Caffeic acid (9.0 mg), ferulic acid (9.7 mg), arabinose (7.5 mg), galactose (9.0 mg), and alanine (4.5 mg) were heated individually, in binary mixtures (CA/Ara, CA/Gal CA/Ala, FA/Ara, FA/Gal FA/Ala) as well as in ternary mixtures (CA/Ara/Ala, CA/Gal/Ala, FA/Ara/Ala, FA/Gal/Ala) at 220 °C in sealed reaction vessels under dry conditions. After different heating times (2.5, 5.0, 7.5, and 10.0 min), the reaction was stopped immediately by cooling the samples to −20 °C in a freezer. The temperature applied for these model roasting experiments were derived from descriptions on coffee roasting, which is commonly deducted at temperatures between 180 and 260 °C.^28^ Additionally, model roasting of hydroxycinnamic acids at 220 °C enabled the synthesis of taste-active compounds, which were identified as key aroma compounds in coffee brews.^29^

Untreated samples (0 min) were prepared as reference. For color measurements, HPLC analyses, and the TEAC assay, the soluble residue was taken up in 1.0 mL of methanol. For GC analysis, a fresh batch of samples was prepared analogously, and the residue was taken up in 1.0 mL of Milli-Q water. Independently of the solvent, the samples were centrifuged (10 min, 8175*g*, 20 °C) after the extraction to remove insoluble solids. Every sample was prepared in triplicate, and all results are given as mean values ± standard deviation. To avoid methylation during HRMS analysis and allow an efficient extraction of the residue, the samples obtained after incubation of the binary and ternary reactions for 5 min were taken up in acetonitrile/water (40/60, v/v).

### Color Measurements

The brown colorants were determined in a semiquantitative approach by measuring the absorbance at 420 nm with a spectrophotometer (UV-1280, Shimadzu Deutschland GmbH, Duisburg, Germany; software: UV Probe Version 2.70). Samples were diluted with methanol when the extinction exceeded 0.9. Browning is given as a color index, calculated as the absorbance at 420 nm and multiplied by the dilution factor per millimole of the reactants (*A*_420_ × *F*/*n*_i_) to allow a comparison between the different reaction systems containing different amounts of reactants. The measurements were performed using a quartz cuvette against methanol.

### HPLC-UV Analysis of Hydroxycinnamic Acids and Heterocyclic Maillard Intermediates

Caffeic acid, ferulic acid, furfural, hydroxymethylfurfural, and pyrrole-2-carbaldeyhde were identified using reference standards. Quantification was performed relative to samples without treatment (0 min). Prior to analysis, the methanolic extracts were diluted in water (1:60) and analyzed by HPLC. For analysis, a Shimadzu analytical HPLC system with the following setup was used: pump, Shimadzu LC20AT; degasser, Shimadzu DGU-20A5; autosampler, Shimadzu SIL-10AF; column, Prodigy ODS-3 C18 (Phenomenex Ltd. Deutschland, Aschaffenburg, Deutschland); column oven, Shimadzu CTO-10AS VP; detector, Shimadzu SPD-20A; software, Shimadzu LabSolutions Version 5.90. The following settings were used: column temperature, 35 °C; flow rate, 0.5 mL/min; eluent A, 0.05% acetic acid in water (v/v); eluent B, methanol; eluent gradient, 0 min, 2.5% B; 10 min, 30% B; 20 min, 65% B; 22 min, 90% B; 26 min, 2.5% B; wavelength for quantification: 270 nm (FF, PA, HMF) and 324 nm (CA, FA).

### Derivatization and GC–MS Analysis of Sugars and Alanine

Alanine, galactose, and arabinose were identified after derivatization using reference standards and quantified relative to the untreated samples (0 min). For derivatization, an aliquot (20 μL) of the aqueous extracts was mixed with an aqueous solution of succinic acid (50 μL, 20 mM, 59.0 mg in 25 mL H_2_O). The solvent was removed under a nitrogen stream and the dry residue was taken up in pyridine (50 μL) and TMCS/BSTFA (50 μL, 1:99, v/v). The samples were incubated at 100 °C for 1 h and the vials were then cooled to −20 °C for 15 min to stop the reaction. After addition of 930 μL of ethyl acetate, the samples were homogenized using a vortex device and measured by GC–MS. For the analysis, a Shimadzu GC system with the following settings was used: GC system, Shimadzu GC-2010; injector, Shimadzu AOC-20i; autosampler, Shimadzu AOC-20s; mass spectrometer, Shimadzu GCMS-QP2010 Plus; column, Agilent Technologies DB-23 (Agilent Technologies Deutschland GmbH, Waldbronn, Deutschland); software, Shimadzu GCMSsolution v2.71. The temperature gradient started at 100 °C with a holding time of 2 min, followed by a temperature increase of 5 °C/min with a final temperature of 200 °C. This temperature was held for 5 min. The instrument settings were as follows: helium was used as carrier gas with a flow of 1.0 mL/min. The injection volume was 1 μL with a split ratio of 1:20. Injection and interface temperature was set to 230 °C, whereas ion source temperature was set to 200 °C. For ionization, a voltage of 70 eV was used. Scan mode from *m*/*z* 28 to 800 was used for the analysis.

### TEAC Assay

The TEAC assay was performed according to Kanzler et al.^30^ with some modifications. The radical stock solution was prepared by mixing an aqueous solution of ABTS (10 mmol/L) 1:1 with an aqueous potassium peroxodisulfate solution (3.5 mmol/L). The stock solution was incubated overnight at room temperature in the absence of light. The working radical solution was prepared by dilution of the stock solution (6:100) with phosphate buffer (50 mmol/L potassium dihydrogen phosphate/hydrogen phosphate, pH = 7.2–7.4). Calibration was performed with six trolox standards (0.01, 0.02, 0.04, 0.06, 0.08, and 0.1 mmol/L; diluted in phosphate buffer). 500 μL of the working solution and 500 μL of the samples (diluted with phosphate buffer) were mixed. Extinction at 734 nm was measured using a Biotek Uvikon XL (Agilent Technologies Inc., Santa Clara, USA) after an incubation time of 120 min. The extinction was multiplied with the dilution factor to allow a comparison of all samples.

### APCI(+) High-Resolution Mass Spectroscopy

HRMS analyses were carried out as described before.^31^ In brief, a Thermo Fisher Scientific Inc. LTQ Orbitrap XL instrument equipped with an Ion Max Source (Waltham, MA, USA) was used. Measurement of the samples was performed via atmospheric pressure chemical ionization in positive ion mode (APCI+) by direct infusion. Reserpine (0.05 mg/mL) was used for mass calibration. The normalized collision energy of collision-induced dissociation was varied from 5 to 50%. For the interpretation of the mass spectra, Freestyle 1.6 (Thermo Fisher Scientific Inc., Waltham, MA, USA) was used.

### Statistical Analysis

All samples were prepared and analyzed in triplicate. All results are shown as means ± standard deviation. Significant differences (*p* < 0.05) were analyzed by two-way analysis of variance (ANOVA) followed by Tukey’s test using the GraphPad Prism 8.0.2 software (San Diego, CA, USA).

## Results and Discussion

Our previous findings revealed that hydroxycinnamic acids are potent browning precursors whose individual treatment results in the formation of colored, melanine-like oligomers and that the reaction with short-chain α-dicarbonyl compounds induces the formation of even more intense, heterogeneous polymers. However, it remains unclear whether these mechanisms can be strictly applied to the reactions contributing to the formation of phenol-containing melanoidins in real food matrices, as the main precursors are sugars and amino compounds. By directly incubating the prominent hydroxycinnamic acids ferulic acid and caffeic acid with sugars and/or alanine, the present study questioned whether these earlier findings are still relevant in “classic” nonenzymatic browning reactions, such as caramelization of individual sugars and the Maillard reaction between reducing sugars and amino compounds in the presence of hydroxycinnamic acids. The temperature of 220 °C applied in these model roasting experiments were derived from descriptions on coffee roasting, which is commonly performed at temperatures between 180 and 260 °C.^28^ Additionally, model roasting of hydroxycinnamiac acids at 220 °C enabled the synthesis of taste-active compounds, which were identified as key aroma compounds in coffee brews.^29^ For evaluation, the color, the conversion of the reactants, the structural composition, and the antioxidant activity of the reaction products formed during the thermal treatment of sugars and/or amino acids in the presence of the hydroxycinnamic acids ferulic acid and caffeic acid were investigated and discussed in the following sections.

### Color Formation

**Figure 1:**
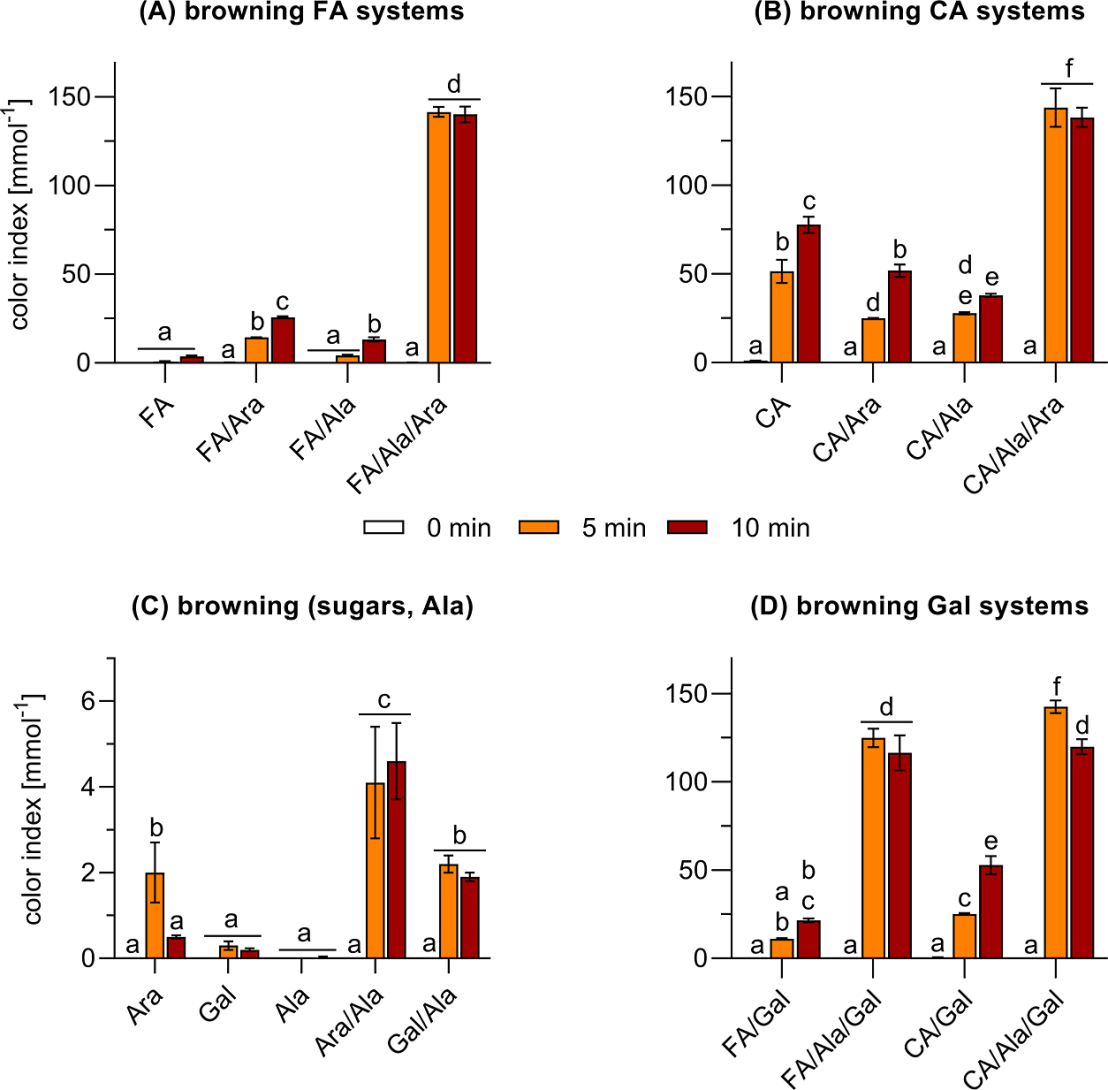
Color formation of individual, binary, and ternary reaction systems composed of different hydroxycinnamic acids (HCA), sugars, and alanine (Ala). Color index of (A) ferulic acid (FA) in combination with arabinose (Ara) and/or alanine, as well as of analogous mixtures of (B) caffeic acid (CA) heated at 220 °C for up to 10 min. Color formation of (C) arabinose, galactose (Gal), alanine, and the corresponding Maillard mixtures Ara/Ala as well as Gal/Ala, and of (D) the phenol-galactose mixtures. Statistical analyses were performed by two-way ANOVA and Tukey’s test (*p* < 0.05). Statistically equal values are designated by equal letters.

To determine the polarity of the colorants formed after incubation of different combinations of hydroxycinnamic acids, sugars, and alanine under roasting conditions, the residues were extracted in methanol and water. In general, the highest yield of colorants resulted for the methanolic extraction, as they were poorly soluble in water. The color formation was analyzed in a semiquantitative approach by measuring the absorbance at 420 nm. The browning potential of the reactants was characterized by the color index (Figure 1), defined as the absorbance of the methanolic extracts per amount of substance used in the corresponding reaction systems. The discussion of the color index allows for a better comparison of the browning potential between the reaction systems by considering the individual amounts of substance of the respective reactants. This enables the interpretation whether a defined combination inhibits, intensifies, or has no effect on the browning observed in comparison to the individual compounds.^22^

In general, the heat-induced color formation of the ferulic acid reaction systems was lower compared to analogous mixtures of caffeic acid, except for the ternary reaction systems. Both, FA/Ara/Ala and CA/Ara/Ala, reached a maximum color index of around 140 mmol^–1^ after 5 min (red bars, Figure 1). Prolonged heating did not result in a significant change of the color soluble in methanol but induced the formation of a dark and poorly soluble residue in the reaction vessels. The formation of such a residue is an indication for the formation of high-molecular weight compounds, in this case: phenol-containing melanoidins. The negligible color index of individually treated FA (4 mmol^–1^, Figure 1A), Ara (2 mmol^–1^, Figure 1C), Ala (0 mmol^–1^, Figure 1C), and Ara/Ala (5 mmol^–1^, Figure 1C) highlight the synergistic browning achieved by the ternary reaction mixtures. A less pronounced, but still significant synergistic effect on the color formation was observed for FA/Ara and FA/Ala. The color index of these binary mixtures was still by multiples higher compared to the sum of the individual compounds. Color increased linearly during heating of FA/Ara and reached around 26 mmol^–1^ after 10 min. Browning was comparatively slower for FA/Ala, but a 5 min heat treatment (orange bar, Figure 1A) resulted in a color index which was comparable to that of FA after 10 min. After 10 min, the color index of FA/Ala increased to approximately 13 mmol^–1^ and was comparable to that of FA/Ara after 5 min of heat treatment.

The color index of pure CA strongly increased in the first reaction period of 5 min to approximately 51 mmol^–1^ and reached about 78 mmol^–1^ after 10 min (Figure 1B). In contrast to the synergistic color formation observed for the binary mixtures of ferulic acid, the browning potential of individually treated CA was higher compared to the binary mixtures CA/Ara and CA/Ala, reaching their highest values at 52 and 38 mmol^–1^ after 10 min, respectively. As a result, the combined incubation of CA/Ara and CA/Alaled to a reduction in the browning potential in comparison to pure CA. Only the incubation of CA/Ara/Ala resulted in a synergistic color formation, suggesting that the browning potential of phenol-containing melanoidins (HCA/Ara/Ala) exceeds that of phenol-containing caramel (HCA/Ara) and melanine-like colorants (pure HCA and HCA/Ala). These findings imply that intermediates exclusively formed in the Maillard reaction, such as nitrogen-containing heterocycles, are vital contributors to color formation. Importantly, a comparable course of browning was observed when Ara was substituted with Gal in the corresponding reaction mixtures of ferulic acid and caffeic acid, which indicates that the reactivity of the reducing sugar is of less relevance compared to the “classic” Maillard reaction (Figure 1D). A complete overview and statistical comparison of the data shown in Figure 1A,B,D can be found in the Supporting Information (Figure S-1).

The higher reactivity of CA compared to FA with regard to their different browning potential was addressed in a previous study.^22^ It was proposed that the differences in color formation and, thus, reactivity between the two hydroxycinnamic acids is attributed to their substitution patterns: the *ortho*-hydroxyl function of caffeic acid exhibits a stronger mesomeric effect compared to the methoxy group of ferulic acid. This is reflected by a higher electron density in the conjugated π-system of caffeic acid compared to ferulic acid, which enables a higher decarboxylation rate of caffeic acid under roasting conditions.^31^ The decarboxylation of these hydroxycinnamic acids was postulated to be a key reaction step preceding the formation of melanin-like colorants formed by the polymerization of the corresponding decarboxylation product.^31^ Further, the high electron density of vinylcatechol (VC), the decarboxylation product of caffeic acid, also results in a higher reactivity to form oligomers and, thus, a higher browning potential in comparison to vinylguaiacol (VG), the decarboxylation product of ferulic acid.^22^

The caramelization of Ara and Gal as well as the Maillard reaction in the systems Ala/Ara and Ala/Gal directly led to the formation of black, insoluble residues and, therefore, a negligible extractable color (Figure 1C). The low solubility of these residues in water and methanol can result from the high temperatures applied in these experiments, promoting pyrolysis and thus the decomposition of the reactants to elemental carbon. Yaylayan & Keyhani^32^ also reported pyrolysis after heating glucose/alanine model systems at 210 °C for 20 s. The high color index of these ternary reaction systems might result from an inhibitory effect of the hydroxycinnamic acids on the pyrolysis observed in the Maillard systems by reacting with the sugars, alanine, and their corresponding degradation products. This is reflected in the formation of soluble, intense brown colorants. Given that the highest browning potential was observed in the ternary reaction mixtures, reactions between the investigated phenolic acids, their corresponding decarboxylation products, and Maillard reaction intermediates are considered to be most efficient for the formation of heterogeneous colorants. Although ferulic acid was found to exhibit a significant lower browning potential than caffeic acid after individual treatment and in the binary mixtures, the comparable browning in the corresponding ternary reaction mixtures of both hydroxycinnamic acids suggests that ferulic acid can indeed contribute to the formation of heterogeneous, intense colorants in complex matrices as present in food.

### Conversion of the Reactants

**Figure 2:**
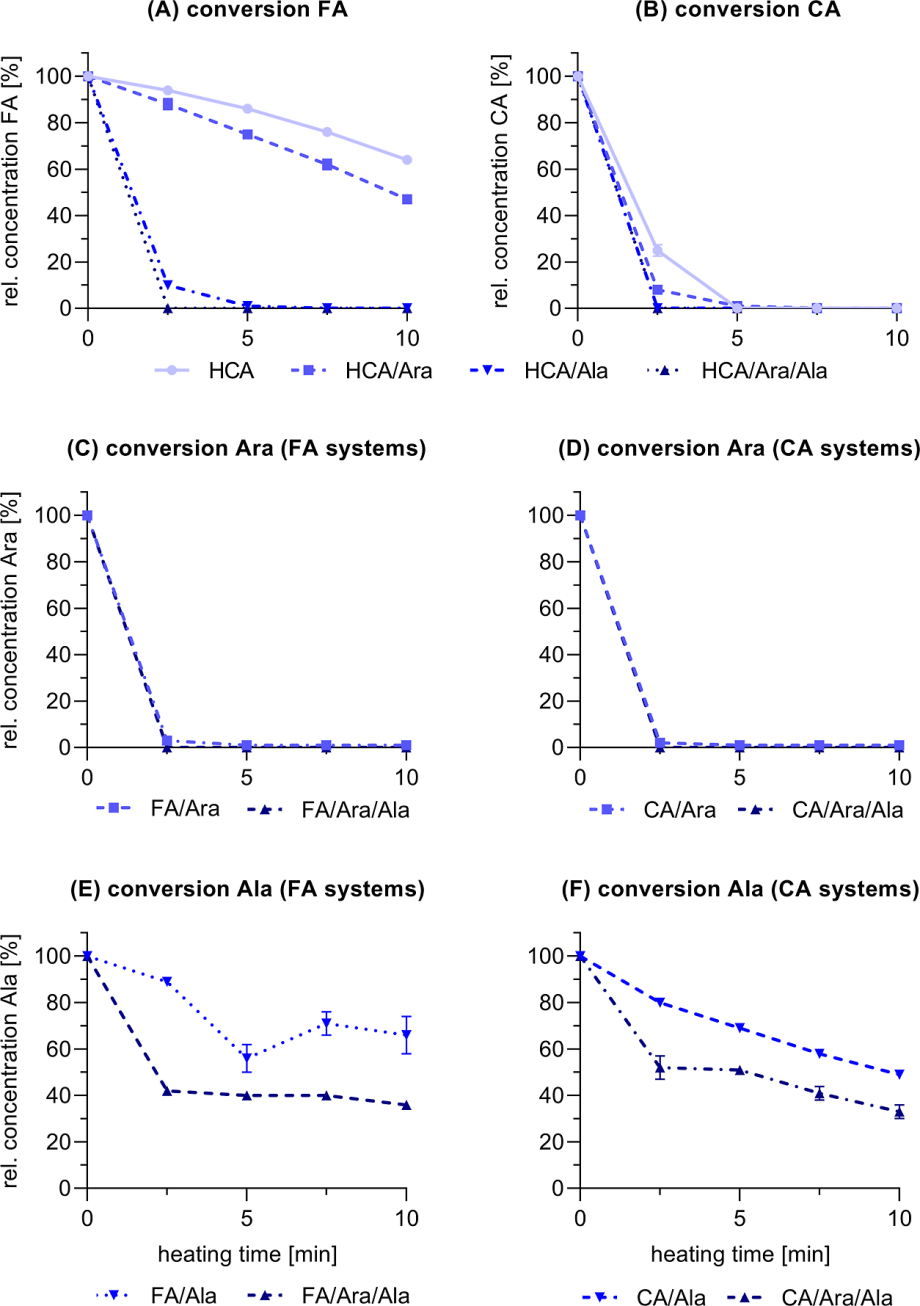
Heat-induced conversion of the hydroxycinnamic acids ferulic acid (FA) and caffeic acid (CA) during the incubation with arabinose (Ara) and/or alanine (Ala) at 220 °C for 10 min. Relative concentration of the reactants (A) FA, (B) CA, (C) Ara (FA systems), (D) Ara (CA systems), (E) Ala (FA systems), and (F) Ala (CA systems) in the corresponding reaction systems. Data obtained for individually treated Ara, galactose (Gal), Ala, the Maillard mixtures of Ara/Ala and Gal/Ala, as well as of the phenol/Gal mixtures are shown in the Supporting Information (Figure S-2).

To gain more information about the role of each reactant in the observed browning reactions, their conversion was monitored. The quantification was performed relative to the concentration in the starting mixture at 0 min (Figure 2). As the heat-induced decarboxylation of hydroxycinnamic acids has been described as a crucial step preceding color formation, the pH value of the methanolic extracts was monitored (Figure S-2) as an indicator for the decarboxylation.^31^

Individual heat treatment of FA induced a linear decline in its concentration to approximately 64% of the initial amount (Figure 2A). This reduction can be primarily attributed to reactions modifying the carboxylic function, as indicated by the increase in the pH value from approximately 3.3 to 4.1 (Figure S-2A). These reactions include decarboxylation and condensation of which decarboxylation reactions were already identified as the key reaction pathway induced by thermal treatment of hydroxycinnamic acids.^22,31^ Consistent with its higher browning potential, the conversion of pure CA was significantly higher compared to ferulic acid. Only around 25% caffeic acid remained after 2.5 min and was fully converted after 5 min (Figure 2B). The conversion of caffeic acid also coincided with a significant increase in the pH value from approximately 4.0 to 5.2, verifying the relevance of decarboxylation reactions (Figure S-2B).

The synergistic browning of FA/Ara coincided with a higher conversion rate of ferulic acid in the binary mixture in comparison to individual FA. Around 47% of the initial amount of ferulic acid was quantified after 10 min, with a pH change comparable to that observed after heat treatment of pure FA (from 3.5 to 4.4, Figure S-2A). Besides decarboxylation of the hydroxycinnamic acid, the heat-induced degradation of sugars yielding short-chain organic acid should be considered when discussing the change in the pH value.^33^ Given that the absolute change in the pH value of FA/Ara was comparable to pure FA and ferulic acid was converted to a higher degree in the binary mixture, the increase in the pH value as induced by the decarboxylation of ferulic acid might be partially offset by the degradation of Ara to short-chain organic acids. Accordingly, arabinose was fully converted after incubation of FA/Ara for 2.5 min (Figure 2C). Therefore, its conversion was significantly faster compared to its individual treatment because around 14% were still available after 2.5 min with subsequent heat treatment resulting in its complete conversion after 5 min (Figure S-3A). Similar trends were observed for CA/Ara: in comparison to pure CA, the conversion rate of caffeic acid was increased and only around 8% of the hydroxycinnamic acid was detected after 2.5 min. Subsequent heating resulted in its complete conversion after 5 min and arabinose was also completely converted after 2.5 min (Figure 2D). The increase in the pH value of CA/Ara was significantly lower compared to pure CA and it only increased in the first 2.5 min of the reaction from around 4.0 to 4.4. It remained at this level until the end of the heating period at 10 min (Figure S-2B). Therefore, the incubation of CA/Ara might have resulted in a higher concentration of free acidic compounds, most likely short-chain acids originating from the heat-induced degradation of arabinose. The higher prevalence of these acids can be explained in two ways. First, the incubation of CA/Ara might result in a significantly higher yield of short-chain acids, which counteract the increase of the pH value as induced by the decarboxylation of caffeic acid. Second, FA/Ara and CA/Ara might yield a comparable amount of short-chain acids, but the reaction products of FA/Ara might be more prone to undergo condensation reactions with these short-chain acids in comparison to those of CA/Ara. However, both pathways imply that different reactions occur during the heat treatment of FA/Ara and CA/Ara.

**Figure 3:**
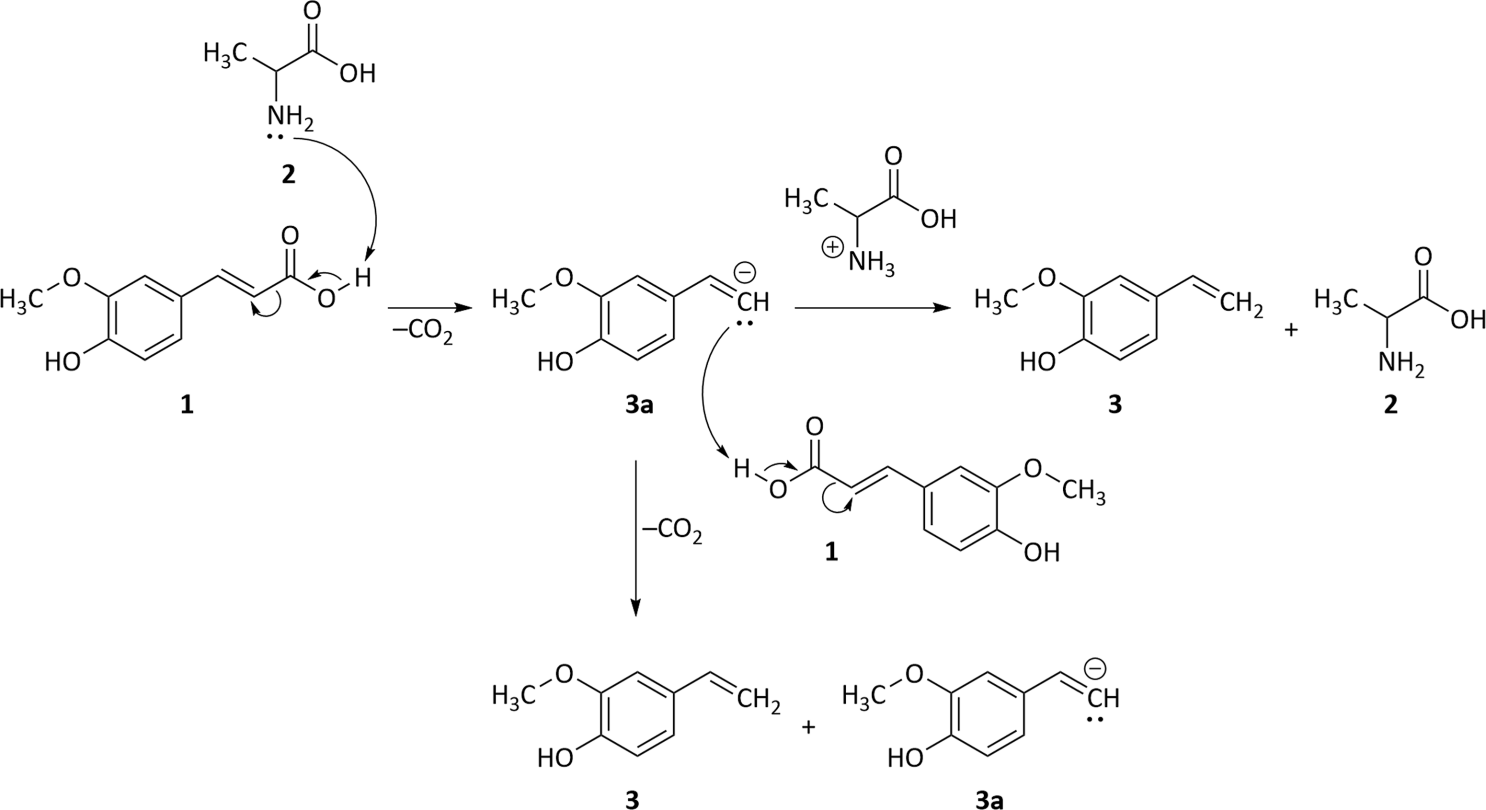
Proposed decarboxylation mechanism of ferulic acid **1** in the presence of alanine **2** to vinylguaiacol **3** via its anion **3a**.

The incubation of ferulic acid in the presence of alanine significantly changed the reaction kinetics: ferulic acid was already converted to around 10% after 2.5 min and was only detected in traces after 5 min of heat treatment. At the same time, the pH value of FA/Ala sharply increased from around 4.9 to 8.1 in the first 5 min and only slightly increased to 8.7 after prolonged heating for 10 min. Given that alanine was only converted to around 89% of its initial amount in the first 2.5 min (Figure 2E), it is assumed that it catalyzed the fast decarboxylation of ferulic acid. This hypothesis is strengthened by the fact that bases like sodium acetate^34^ and/or free amines such as butylamine^35^ are commonly used as catalysators for the decarboxylation of hydroxycinnamic acids. However, proteinogenic amino acids like alanine have not been considered as catalysators for the decarboxylation of hydroxycinnamic acids in food, so far. A proposed mechanism for the accelerated decarboxylation of ferulic acid **1** in the presence of alanine **2** is discussed in the following (Figure 3): in the first step of the reaction, the free electron pair of the amino function of alanine **2** abstracts the acidic proton of ferulic acids’ **1** carboxylic function. This induces the decarboxylation of ferulic acid **1** to the vinylguaiacol anion **3a**. The negative charge of **3a** is not stabilized by resonance; thus, **3a** is considered as a highly reactive intermediate. Its neutralization may either be achieved by abstracting the proton from the alanine cation, yielding vinylguaiacol **3** and alanine **2**. Alternatively, **3a** may react as a base inducing the decarboxylation of another unit of ferulic acid **1**, yielding vinylguaiacol **3** and its anion **3a**. Both reaction mechanisms yield vinylguaiacol **3** and a base that could initiate the decarboxylation of another ferulic acid molecule, resulting in a highly accelerated conversion of ferulic acid as observed in the presence of alanine. It is to note that **3a** might also perform an intermolecular proton shift with the hydroxy proton of another unit **3a** or **3**, yielding a stabilized phenolate-ion. However, as long as ferulic acid **1** or protonated alanine are available in the mixture, which are considered to be stronger acids, this pathway seems less likely.

After ferulic acid was completely converted, the relative concentration of alanine declined to approximately 65% after 5 min and its concentration did not change significantly afterward. Consequently, the reactivity of alanine was dependent on the concentration of ferulic acid: in the first stage of the reaction, it promoted the decarboxylation of ferulic acid. In the second stage of the reaction, it might have reacted with the degradation products of ferulic acid, such as its decarboxylation product vinylguaiacol and oligomers thereof, whereas these reactions did not strongly contribute to the formation of colored products (Figure 1A). For an increase in the color intensity, an enlargement of the conjugated π-electron system of the corresponding reaction products would be required, which is not considered to result from the reactions between vinylguaiacol and alanine to a significant extent. However, the reactivity of alanine, as indicated by its conversion, was significantly increased in the presence of ferulic acid, because its individual treatment did not result in a change in its concentration (Figure S-3B).

The generally high conversion rate of caffeic acid as observed for CA and CA/Ara was further accelerated in the presence of alanine. This was reflected by the complete conversion of caffeic acid after 2.5 min. This conversion was also accompanied by an increase in the pH value of CA/Ala from approximately 5.1 to 8.2, which strengthens the hypothesis that alanine might indeed promote the decarboxylation of the investigated hydroxycinnamic acids. In contrast to FA/Ala, heat treatment of CA/Ala induced a linear decline in the concentration of alanine to approximately 49% of the initial amount (Figure 2F). The higher conversion rate of alanine could result from the higher reactivity of caffeic acid and its corresponding decarboxylation product vinylcatechol. The more reactive intermediates resulting from the conversion of caffeic acid might be more prone to undergo subsequent reactions with alanine, resulting in a higher conversion compared to that observed for FA/Ala. One example is the Strecker-like degradation of alanine that is only possible after oxidation of caffeic acid or vinylcatechol to their corresponding *ortho*-quinones. However, such reactions are not considered to significantly contribute to color formation, which was also reflected by the decreased browning potential of CA/Ala in comparison to pure CA (Figure 1B).

The hydroxycinnamic acid (Figure 2A,B) and Ara (Figure 2C,D) in FA/Ara/Ala and CA/Ara/Ala were completely converted after 2.5 min. Regarding alanine, its initial conversion to approximately 42 and 52% after 2.5 min was higher for FA/Ara/Ala (Figure 2E) in comparison to CA/Ara/Ala (Figure 2F), respectively. The reaction kinetic significantly differed as the concentration of alanine remained constant after the initially faster declined observed for FA/Ara/Ala, whereas it was linearly converted in the mixture of CA/Ara/Ala to about 33% after 10 min. Initially, for both mixtures, the highest increase in the pH value correlated with the complete conversion of the corresponding hydroxycinnamic acid after 2.5 min with the pH of FA/Ara/Ala increasing from 4.7 to 6.3 (Figure S-2A) and that of CA/Ara/Ala from 5.1 to 6.1 (Figure S-2B). In the following reaction period, the pH value of both ternary mixtures significantly increased to about 7.1, which shows another difference to the binary reaction systems. Besides the decarboxylation of the hydroxycinnamic acid, the intermediary formation of α-dicarbonyl compounds and/or *ortho*-quinones could induce the decarboxylation of alanine by a Strecker degradation. For CA/Ara/Ala, this is reflected by the continuous conversion of alanine and the parallel increase of the pH value. As the concentration of Ala did not significantly decline after 2.5 min for FA/Ara/Ala, the decarboxylation of alanine cannot be considered to result in the observed increase of the pH value. Instead, follow-up reactions with intermediary formed short-chain acids from the degradation of Ara are proposed to contribute to the increase of the pH value observed in the second stage of the reaction.

Generally, the substitution of arabinose with galactose in the respective reaction systems led to similar observations in both the change in pH value (Figure S-2) and reaction kinetics (Figure S-3B–D), with the exception that the relative concentration of alanine was significantly lower after heat treatment of Ara/Ala (around 30%) in comparison to Gal/Ala (around 50%, both shown in Figure S-3B). However, the conversion of both sugars (Figure S-3A) and alanine (Figure S-3B) in the binary Maillard mixtures (Ara/Ala and Gal/Ala) was comparable to the ternary mixtures with caffeic acid or ferulic acid, respectively. Further details can be found in the Supporting Information. Although these sugars exhibit different reactivities and yield different reactive intermediates, this does not significantly affect their conversion, the change in the pH value, and the color formation observed in the corresponding reaction system.

### Formation of Heterocyclic Maillard Intermediates

**Figure 4:**
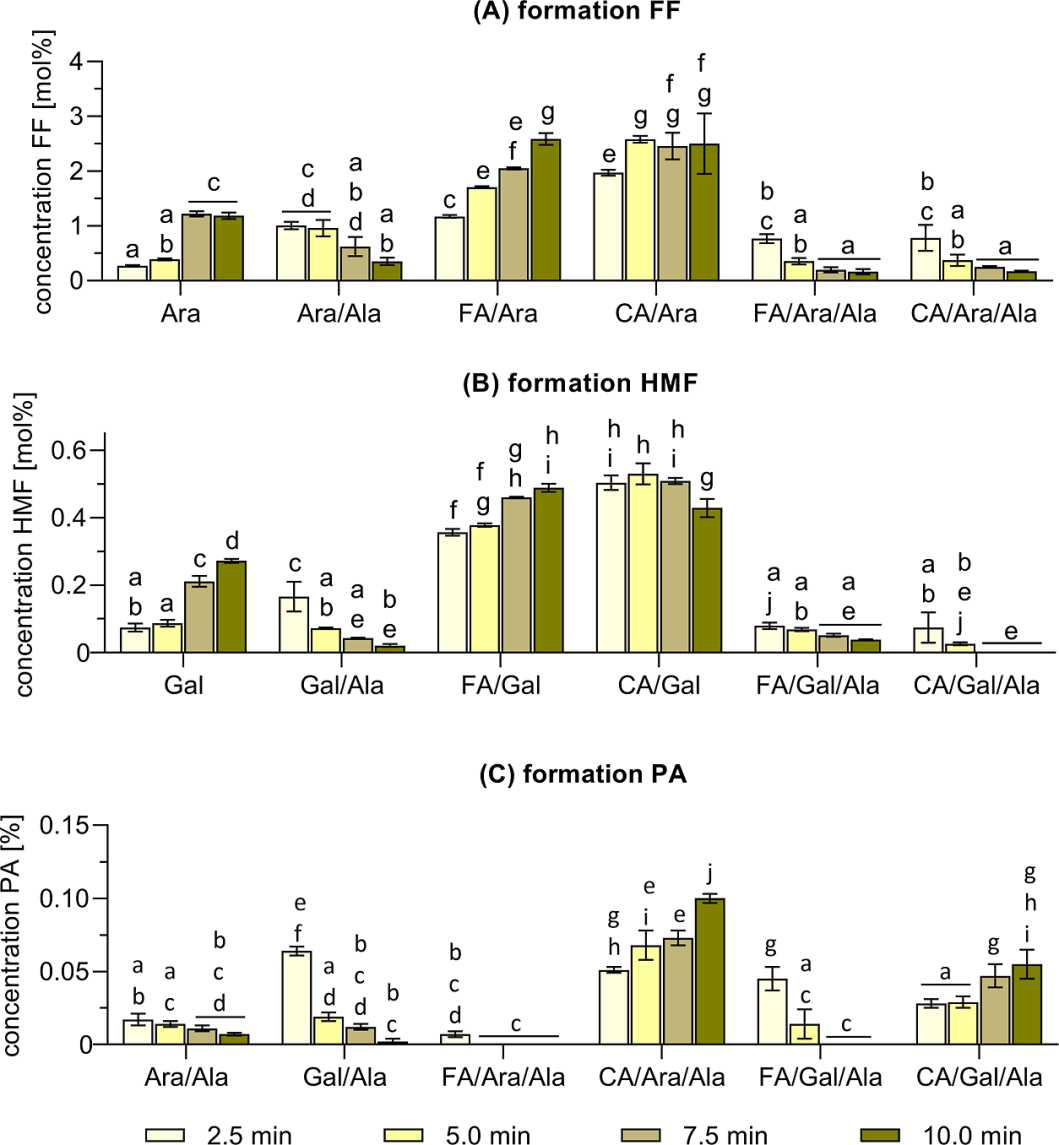
Heat-induced formation of the heterocyclic Maillard intermediates furfural (FF), hydroxymethylfurfural (HMF), and pyrrole-2-carbaldehyde (PA). Formation of (A) FF, (B) HMF, and (C) PA after individual incubation of arabinose (Ara) and galactose (Gal) as well as in combination with alanine (Ala) and the hydroxycinnamic acids ferulic acid (FA) or caffeic acid (CA). Statistical analyses were performed by two-way ANOVA and Tukey’s test (*p* < 0.05). Statistically equal values are designated by equal letters.

Given that the ternary reaction systems exhibited the highest color formation, we hypothesized that the reactions between phenolic compounds and reactive Maillard intermediates are key contributors to color formation. Therefore, we monitored the formation of prominent heterocyclic color precursors in nonenzymatic browning reactions, namely, FF, HMF, and PA, in the carbohydrate-containing reaction systems (Figure 4). It is noteworthy that the furan derivatives FF and HMF are also relevant intermediates in the caramelization of pure sugars. However, their formation is promoted by the catalytic effect of amines.^36^ Nitrogen-containing heterocyclic compounds like PA, which exhibit an even higher browning potential as furans,^37^ are exclusively formed in the Maillard reaction.^36,38^

Heat treatment of Ara resulted in a maximum FF concentration of approximately 1.2 mol % after 7.5 min (Figure 4A). In contrast, incubation of FA/Ara and CA/Ara led to a 2-fold higher FF concentration, reaching around 2.5 mol % after 10 and 5 min, respectively. This suggests that the presence of both hydroxycinnamic acids promoted FF formation. However, prolonged heating did not result in a significant change in the concentration of FF, indicating a reduced reactivity in subsequent browning reactions. A different kinetic relationship was observed in Ara/Ala, FA/Ara/Ala, and CA/Ara/Ala, with the concentration of FF reaching a maximum of around 0.8 mol % (HCA/Ara/Ala) and 1.0 mol % (Ara/Ala) after a 2.5 min heat treatment. Subsequent heat treatment led to a decline in FF to approximately 0.4 mol % (Ara/Ala) and 0.2 mol % (HCA/Ara/Ala), with a faster and more pronounced decrease in the ternary reaction systems. This indicates that the reactivity of FF to undergo subsequent browning reactions is strongly dependent on the chemical environment and that the presence of nitrogen-containing compounds (here Ala) is key for the carbonyl intermediates to subsequently contribute to color formation.

Similar findings were observed for the formation of HMF in the reaction systems of Gal (Figure 4B). Individual treatment of Gal resulted in a linear increase in the HMF concentration to about 0.3 mol % after 10 min. The presence of hydroxycinnamic acids promoted the formation of HMF, which was reflected by a maximum concentration of around 0.5 mol % after 5 min (CA/Gal) and 10 min (FA/Gal). After 10 min, the HMF concentration slightly decreased in CA/Gal, indicating its involvement in the formation of colored reaction products. Incubation of the Maillard mixture (Gal/Ala) and the ternary reaction systems (HCA/Gal/Ala) led to an initial spike in the HMF concentration to approximately 0.2 and 0.1 mol % at 2.5 min, respectively. Subsequent heat treatment resulted in a significant decline of HMF, with only traces being detected after 10 min. Generally, these findings imply a higher contribution of HMF to the formation of phenol-containing caramels in the mixture of CA/Gal in comparison to FA/Gal. Again, the presence of Ala significantly promoted the conversion of HMF, presumably resulting in the formation of phenol-containing melanoidins. Considering the browning potential of these ternary reaction systems (Figure 1B,C), it becomes apparent that the declining concentration of HMF correlates with an increase in the browning intensity. This demonstrates that under the roasting conditions applied in this study, ferulic acid and caffeic acid do not inhibit the propagation of the Maillard reaction by trapping intermediary carbonyl compounds, but they contribute to an increased browning intensity.

The increased formation of furan derivatives, induced by the combined incubation of sugars and hydroxycinnamic acids (or derivatives thereof), has been described previously. However, no concise explanation was provided for these findings.^39,40^ The herein observed increase in the formation of FF (in HCA/Ara) and HMF (in HCA/Gal) compared to pure Ara and Gal might be attributed to the acidity of these hydroxycinnamic acids. In carbohydrate chemistry, an acidic environment is known to catalyze the dehydration and cyclization of reducing sugars.^41^ This results in higher yields of the corresponding heterocyclic intermediates, as reflected by the present findings. Consequently, ferulic acid and caffeic acid may act as catalysators during sugar degradation due to their acidity. This thesis is verified by the correlation between the concentration of the respective hydroxycinnamic acid and the formation of heterocyclic compounds observed herein: ferulic acid is still available until the end of the investigated reaction period, which coincides with a linear increase of FF and HMF (Figure 4A,B). However, caffeic acid is completely converted after 5 min. This corresponds with the FF and HMF concentration reaching its maximum in the respective reaction mixtures. However, to allow a general conclusion of the assumed catalytic effect of hydroxycinnamic acids on the conversion of sugars, a higher number of different sugars should be investigated.

Besides the furans FF and HMF, the formation of low amounts of PA was detected in the binary and ternary reaction mixtures containing a carbohydrate and Ala (Figure 4C). The maximum PA concentration in the reaction mixtures of Ara/Ala (0.01 mol %), Gal/Ala (0.06 mol %), FA/Ara/Ala (<0.01 mol %), and FA/Gal/Ala (0.05 mol %) was detected after heat treatment for 2.5 min. Subsequent heat treatment resulted in an (almost) complete conversion of PA, and only traces were detected after 10 min in Ara/Ala and Gal/Ala. PA was no longer detectable in FA/Ara/Ala and FA/Gal/Ala. In contrast, a continuous increase in its concentration was observed in CA/Ara/Ala and CA/Gal/Ala, with its maximum concentration reaching 0.1 and 0.06 mol %, respectively. Therefore, caffeic acid significantly promoted the formation of PA in comparison to the binary sugar-amino acid mixture. Although PA is primarily considered as a reaction product formed in the Maillard reaction of pentoses,^42^ its exact formation mechanism is not sufficiently described to date. However, we detected higher amounts of PA in selected Gal-containing reaction systems in comparison to analogous ones of Ara (Ara/Ala vs Gal/Ala; FA/Gal/Ala vs FA/Ara/Ala). As heat treatment can induce the degradation of Gal into reactive C_5_-intermediates,^43^ this pathway seems to be favored in the presence of ferulic acid. On the other hand, formation of PA is significantly higher when caffeic acid is added to a mixture of Ara/Ala. In general, these findings provide important indications that the general mechanism preceding the formation of heterogeneous colorants in the reaction systems of ferulic acid significantly differs from those of caffeic acid. Further, PA is a nitrogen-containing, electron-rich intermediate that could play a vital role in the formation of phenol-containing melanoidins. It might contribute to cross-linking reactions due to its ambivalent reactivity as a donor and acceptor in nucleophilic reactions.

### Compositional Elucidation of Colored Reaction Products

The structural composition of the colored reaction products was investigated by HRMS. This approach has been established as a valuable method especially for complex mixtures, as the mass accuracy allows the assignment to specific sum formulas.^37,44,45^ To interpret the signals detected in the investigated reaction mixtures, the reactants and their primary degradation products were considered for a structure assignment. This includes VC from CA, VG from FA, FF from Ara or Gal, PA from Ara or Gal and Ala, and HMF from Gal as well as combinations thereof. Furthermore, condensation reactions (−*n* × H_2_O) and oxidation (−*n* × H_2_) were identified as key reactions in nonenzymatic browning and therefore also included in the assignment.^46,47^Table 1 shows selected signals with a relative intensity of at least 1% detected in the reaction mixtures of CA/Ara, CA/Ala, CA/Ara/Ala, FA/Ara, FA/Ala, and FA/Ara/Ala.

**Table 1:** Assignment of Selected Signals to Reaction Products Detected by APCI(+)-HRMS Analysis of the Reaction Mixtures Composed of CA/Ara, CA/Ala, CA/Ara/Ala, FA/Ara, FA/Ala, and FA/Ara/Ala after Heat Treatment at 220 °C for 5 min^a^

	structure assignment									
reaction mixture	compounds			H_2_O	H_2_	composition	exp. *m*/*z*	theo. *m*/*z*	rel. error (ppm)	rel. int. [%]
FA/Ara	1 × VG			0	0	C_9_H_10_O_2_H^+^	151.0753	151.0754	–0.6	9
	1 × FA			–1	0	C_10_H_8_O_2_H^+^	177.0543	177.0546	–1.6	16
	1 × FA			0	0	C_10_H_10_O_3_H^+^	195.0649	195.0652	–1.3	100
	1 × VG	1 × MGO		0	–1	C_12_H_12_O_4_H^+^	221.0808	221.0808	–0.4	3
	1 × VG	1 × FF		0	0	C_14_H_14_O_4_H^+^	247.0964	247.0965	–0.4	2
	1 × FA	1 × VG		0	0	C_19_H_20_O_6_H^+^	345.1332	345.1333	–0.3	2
	1 × VG			0	0	C_9_H_10_O_2_H^+^	151.0753	151.0754	–0.6	9
										
CA/Ara	1 × VC			0	0	C_8_H_8_O_2_H^+^	137.0594	137.0597	–1.9	99
	1 × VC	1 × FF		–1	0	C_13_H_10_O_3_H^+^	215.0700	215.0703	–1.3	2
	1 × VC	1 × FF		0	0	C_13_H_12_O_3_H^+^	233.0806	233.0808	–1.1	6
	1 × VC	1 × Ara		–2	0	C_13_H_14_O_5_H^+^	251.0911	251.0914	–1.2	3
	1 × VC	1 × FF	1 × MGO	–2	0	C_16_H_12_O_4_H^+^	269.0805	269.0808	–1.1	13
	2 × VC			0	0	C_16_H_16_O_4_H^+^	273.1118	273.1121	–1.2	4
	1 × VC	1 × FF	1 × MGO	–1	0	C_16_H_14_O_5_H^+^	287.0911	287.0914	–1.2	4
	1 × VC	2 × FF		0	0	C_18_H_16_O_6_H^+^	329.1015	329.1020	–1.3	3
	2 × VC	1 × FF		–1	0	C_21_H_18_O_5_H^+^	351.1223	351.1227	–1.1	3
	2 × VC	1 × FF		0	–1	C_21_H_18_O_6_H^+^	367.1172	367.1176	–1.1	3
	2 × VC	1 × FF		0	0	C_21_H_20_O_6_H^+^	369.1329	369.1333	–1.0	12
	2 × VC	1 × Ara		–2	–1	C_21_H_20_O_7_H^+^	385.1277	385.1282	–1.2	1
	2 × VC	1 × Ara		–2	0	C_21_H_22_O_7_H^+^	387.1433	387.1438	–1.5	6
	2 × VC	1 × Ara		–2	–1	C_21_H_22_O_8_H^+^	403.1381	403.1387	–1.7	1
	2 × VC	1 × FF	1 × MGO	–2	0	C_24_H_20_O_6_H^+^	405.1326	405.1333	–1.7	26
	3 × VC			0	0	C_24_H_24_O_6_H^+^	409.1639	409.1646	–1.7	2
										
FA/Ala	1 × VG			0	0	C_9_H_10_O_2_H^+^	151.0753	151.0754	–0.7	100
	1 × VG	1 × Ala	–1	–1	0	C_12_H_15_O_3_NH^+^	222.1124	222.1125	–0.4	2
	1 × VG	1 × Ala		0	0	C_12_H_13_O_2_NH^+^	240.1230	240.1230	–0.1	1
	2 × VG			0	–1	C_18_H_18_O_4_H^+^	299.1277	299.1278	–0.3	5
	2 × VG			0	0	C_18_H_20_O_4_H^+^	301.1433	301.1434	–0.3	6
	2 × VG	1 × Ala		–1	0	C_21_H_25_O_5_NH^+^	372.1805	372.1805	–0.1	1
	3 × VG	1 × Ala		0	0	C_30_H_37_O_8_NH^+^	540.2590	540.2592	–0.5	4
	4 × VG	1 × Ala		0	0	C_39_H_45_O_10_NH^+^	688.3116	688.3116	–0.1	2
	5 × VG	1 × Ala		0	0	C_48_H_57_O_12_NH^+^	840.3954	840.3954	0.1	1
										
CA/Ala	1 × VC			0	0	C_8_H_8_O_2_H^+^	137.0594	137.0597	–1.9	99
	2 × VC			0	–3	C_16_H_10_O_4_H^+^	267.0649	267.0652	1.0	4
	2 × VC			0	–2	C_16_H_12_O_4_H^+^	269.0805	269.0808	–1.2	29
	2 × VC			0	–1	C_16_H_14_O_4_H^+^	271.0960	271.0965	–1.8	100
	2 × VC	1 × Ala		0	–2	C_19_H_19_O_6_NH^+^	358.1280	358.1285	–1.3	1
	3 × VC			0	–3	C_24_H_18_O_6_H^+^	403.1169	403.1176	–1.8	6
	3 × VC			0	–2	C_24_H_20_O_6_H^+^	405.1326	405.1333	–1.8	21
	1 × VC			0	0	C_8_H_8_O_2_H^+^	137.0594	137.0597	–1.9	99
	2 × VC			0	–3	C_16_H_10_O_4_H^+^	267.0649	267.0652	1.0	4
	2 × VC			0	–2	C_16_H_12_O_4_H^+^	269.0805	269.0808	–1.2	29
	2 × VC			0	–1	C_16_H_14_O_4_H^+^	271.0960	271.0965	–1.8	100
	2 × VC	1 × Ala		0	–2	C_19_H_19_O_6_NH^+^	358.1280	358.1285	–1.3	1
	3 × VC			0	–3	C_24_H_18_O_6_H^+^	403.1169	403.1176	–1.8	6
	3 × VC			0	–3	C_24_H_18_O_6_H^+^	403.1169	403.1176	–1.8	6
										
FA/Ara/Ala	1 × VG			0	0	C_9_H_10_O_2_H^+^	151.0752	151.0754	–0.9	100
	1 × VG	1 × Ala		0	0	C_12_H_13_O_2_NH^+^	240.1229	240.1230	–0.6	2
	2 × VG			0	0	C_18_H_20_O_4_H^+^	301.1433	301.1434	–0.5	2
	2 × VG	2 × Ala	1 × FF	–3	0	C_29_H_32_O_7_NH^+^	521.2282	521.2282	0.0	1
	2 × VG	2 × Ala	1 × PA	–1	0	C_31_H_39_O_12_NH^+^	646.2612	646.2607	–0.8	1
										
CA/Ara/Ala	1 × VC			0	0	C_8_H_8_O_2_H^+^	137.0595	137.0597	–1.4	44
	1 × VC	1 × Ala		0	0	C_11_H_15_O_4_NH^+^	226.1072	226.1074	–0.9	4
	1 × VC	1 × PA		0	0	C_13_H_13_O_3_NH^+^	232.0965	232.0968	–1.3	5
	1 × VC	1 × FF		0	0	C_13_H_14_O_4_H^+^	235.0963	235.0965	–0.9	4
	2 × VC			0	–1	C_16_H_14_O_4_H^+^	271.0962	271.0965	–1.0	87
	1 × VC	1 × Ala	1 × FF	0	0	C_16_H_19_O_6_NH^+^	322.1284	322.1285	–0.5	2
	1 × VC	1 × PA	1 × FF	0	0	C_18_H_17_O_5_NH^+^	328.1176	328.1179	–1.1	2
	1 × VC	1 × Ara	1 × PA	–2	0	C_18_H_19_O_6_NH^+^	346.1280	346.1285	–1.5	1
	2 × VC	1 × FF		–1	0	C_21_H_18_O_5_H^+^	351.1226	351.1227	–0.4	2

a Only signals with a relative intensity of at least 1% and a relative error below 5 ppm were considered for the assignment. For the structural assignment, the reactants ferulic acid (FA), caffeic acid (CA), arabinose (Ara), and alanine (Ala) and common conversion products, such as hydroxymethylfurfural (HMF), furfural (FF), pyrrole-2-carbaldehyde (PA), vinylcatechol (VC), and vinylguaiacol (VG) were considered. Data obtained for the corresponding galactose reaction systems are shown in the Supporting Information (Table S-1).

In general, the data obtained by HRMS confirmed the pivotal role of decarboxylation in the nonenzymatic browning of the investigated hydroxycinnamic acids: the obtained signals can be primarily assigned to the respective decarboxylation products, vinylcatechol (VC) and vinylguaiacol (VG), as well as reaction products thereof. Caffeic acid was not detected in its native form nor as a constituent of the assigned products. In contrast, the lower reactivity of ferulic acid was reflected in its presence as the base peak in the reaction mixtures of FA/Ara (Table 1, Figure S-4A) and FA/Gal (Table S-1, Figure S-5A). However, vinylguaiacol and heterogeneous products composed of vinylguaiacol and degradation products of the corresponding sugar were also detected in these mixtures (e.g., *m*/*z* 221: VG + MGO – H_2_; *m*/*z* 247 VG + FF).

Regarding CA/Ara (Figure S-4B), the extent of signals assigned to heterogeneous phenol-carbonyl products was higher compared to FA/Ara. Apart from vinylcatechol (*m*/*z* 137), its dimer (*m*/*z* 273), and its trimer (*m*/*z* 406), the majority of the signals were assigned to heterogeneous products composed of vinylcatechol and degradation products of Ara, such as (di)deoxypentosone, FF, and MGO. As all of these degradation products are considered as electrophilic carbonyls, the products are proposed to be formed by electrophilic aromatic substitution reactions with vinylcatechol as the nucleophile. Among these, both adducts and condensation products were detected, for example: *m*/*z* 233 (VC + FF), *m*/*z* 269 (VC + FF + MGO – 2 × H_2_O), *m*/*z* 329 (VC + 2 × FF), and *m*/*z* 351 (2 × VC + FF). The high prevalence of condensation products can be explained by the enlargement of the conjugated π-electron system that results from the dehydration. Additionally, these conjugated products may contribute to the color observed in the reaction mixture.

The presence of Ala notably accelerated the conversion of ferulic acid in FA/Ala (Figure 2B) even though this did not result in an intense browning. Consequently, exclusively signals assigned to vinylguaiacol (*m*/*z* 151) and related products, including its dimer (*m*/*z* 299 (−H_2_), *m*/*z* 301), and heterogeneous oligomers consisting of up to five units of VG and one unit of Ala (*m*/*z* 840), were detected (Table 1, Figure S-4C). This verifies that Ala indeed facilitated the decarboxylation of ferulic acid. However, these oligomers do not seem to contribute to the color of the reaction mixtures.

Given that the presence of Ala increased the conversion rate of caffeic acid, the amine was proposed to accelerate the heat-induced decarboxylation of caffeic acid even more (Figure 2B). However, only one signal *m*/*z* 358 (2 × VC + Ala −2 × H_2_) in the HRMS spectrum of CA/Ala (Figure S-4D) can be assigned to an adduct of vinylcatechol and Ala. The rest of the signals were assigned to vinylcatechol and different redox stages of its di- and trimer. In comparison to individually treated CA (Figure 1A), the browning potential per mole of the reactants was reduced by the equimolar addition of Ala. This indicates that even though the decarboxylation of the hydroxycinnamic acid might be catalyzed by Ala, the subsequent color formation of vinylcatechol was impaired. The low browning potential obtained after incubation of FA/Ala and CA/Ala can be explained by the reactivity of Ala: its side chain, a methyl group, can be considered as comparatively inert. Thus, the amino group and the carboxylic moiety of alanine are the only reactivity centers to be considered to enable a reaction with the investigated phenolic acids and their corresponding decarboxylation products. The amino group is known to react as a nucleophile with a quinone formed after oxidation of a phenol.^48^ However, these Michael-like products are not considered as colorants as the extent of the conjugated π-electron system is reduced by this reaction. Another type of reaction occurring between amino acids and phenolic compounds are condensation reactions. For example, the condensation between the carboxylic moiety of caffeic acid and the amino group of Ala results in the formation of an amide.^49^ Esterification can be achieved by the reaction of the carboxylic group of alanine and one hydroxy group of an aromatic hydroxy moiety. Another type of condensation can be also initiated by the nucleophilic addition of the nitrogen atom of the amino group to the carbonyl group of a quinone, subsequently leading to elimination of water, which can be interpreted as the first step of an Strecker-like reaction.^50,51^ Again, these types of condensation reactions with Ala are not considered to result in a sufficient enlargement of the conjugated system to yield colored products.

By investigating the ternary reaction systems, we discovered that PA, which was identified as a Maillard intermediate in these reaction mixtures, formed heterogeneous products with vinylguaiacol (*m*/*z* 646:2 × VG + 2 × Ala + PA, Figure S-4E) and vinylcatechol (*m*/*z* 232: VC + PA, Figure S-4F). The formation of such products can be explained through electrophilic aromatic substitution reactions, in which vinylguaiacol, vinylcatechol, and PA can react as both donor and acceptor. Apart from the electron-rich aromatic systems present in all three compounds, PA can react as an acceptor with its electrophilic carbonyl group. Both vinylguaiacol and vinylcatechol exhibit reduced electron density at the C-1 position of the vinyl group, suggesting the possibility of a nucleophilic attack at this particular position. Further, other heterogeneous reaction products of VC and VG were detected in the corresponding ternary systems. Specifically, *m*/*z* 322 (VC + Ala + FF), *m*/*z* 328 (VC + PA + FF), and *m*/*z* 346 (VC + Ara + PA – 2 × H_2_O) were identified in CA/Ara/Ala as well as *m*/*z* 521 (2 × VG + 2 × Ala + FF) in FA/Ara/Ala. In summary, the reaction products formed in these ternary mixtures exhibit a higher molecular weight compared to those in the binary mixtures which coincided with the highest browning among the investigated reaction systems (Figure 1). We propose that nitrogen-containing aromatic compounds formed in the Maillard reaction of Ara/Ala and Gal/Ala, such as PA, are a vital contributor to the formation of the intense colorants. Due to their ambivalent reactivity and conjugated π-electron system, it was assumed that the cross-linking of PA, vinylcatechol, and vinylguaiacol significantly contributes to an increase in the molecular weight and the browning intensity of the resulting reaction products. Consequently, the synergistic browning observed in the ternary reaction systems is a result of the polymerization of phenolic compounds with heterocyclic Maillard reaction products, such as FF, PA, and HMF. The intense color formation likely results from cross-linking and condensation reactions that significantly enlarge the π-electron system and thus facilitate the formation of large chromophores.

Similar findings were observed in reaction systems containing Gal instead of Ara (Table S-1 and Figure S-5). The primary difference was the higher prevalence of HMF compared to FF in the data obtained by HRMS. This aligns with FF being the common degradation product in nonenzymatic browning of pentoses, whereas HMF is characteristic for the degradation of hexoses.^36^ However, FF can also be formed by thermally induced degradation of Gal via several reaction steps^43^ and directly by the cleavage of HMF.^41^

### Antioxidant Properties

**Figure 5:**
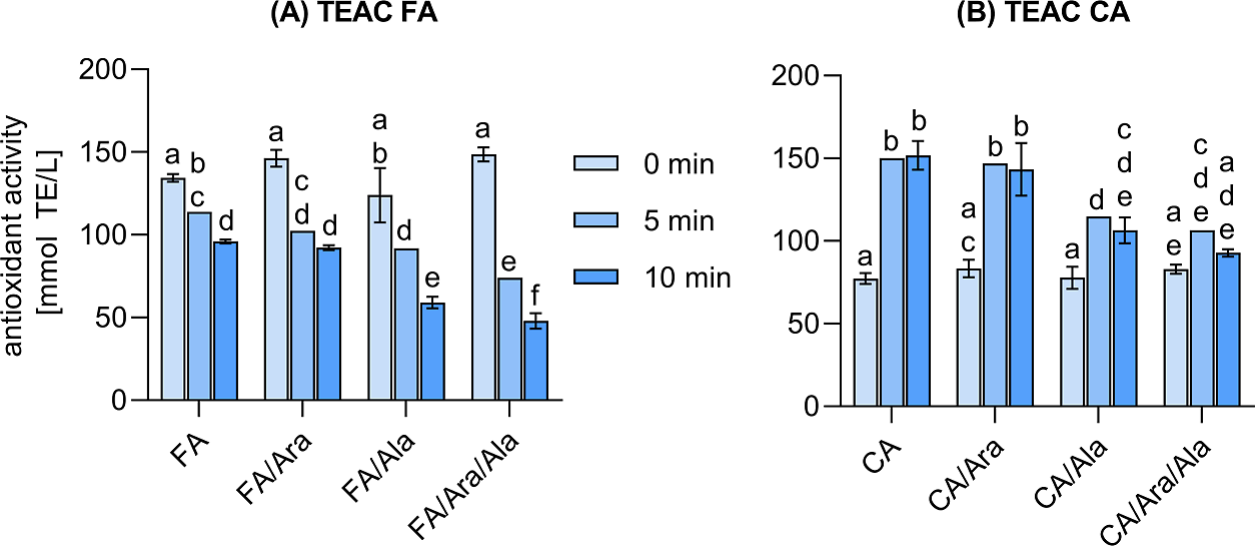
Change in color and antioxidant activity after heating under roasting conditions for (A) ferulic acid (FA) and (B) caffeic acid (CA). Statistical analyses were performed by two-way ANOVA and Tukey’s test (*p* < 0.05). Statistically equal values are designated by equal letters. Data obtained for individually treated arabinose (Ara), galactose (Gal), alanine (Ala), the Maillard mixtures of Ara/Ala and Gal/Ala as well as of the HCA/Gal mixtures are shown in the Supporting Information (Figure S-6).

Maillard reaction products, in particular, reductones, reductone ethers,^30^ and melanoidins,^52^ are characterized as potent antioxidants. Therefore, nonenzymatic browning does not only influences the color, taste, or aroma of food, but it also contributes to the oxidative stability and thus, a longer shelf life of processed foods.^53^ For “classic” melanoidins, which are considered as products primarily derived from carbohydrates and amino compounds, studies have shown that their antioxidant properties correlate with their browning intensity. Therefore, melanoidins exhibiting a high browning intensity were also found to be strong antioxidants.^54,55^ In contrast, the highest antioxidant activity of phenol-containing melanoidins obtained from roasted coffee was attributed to low-molecular-weight compounds, which exhibited lower color intensity than the products with a high molecular weight.^56,57^ The differences between these two types of melanoidins imply that the physicochemical properties of such products strongly depend on the reactants involved in their formation. Nevertheless, phenol-containing melanoidins should be considered as important antioxidants in human nutrition, which is also reflected in empirical data showing that melanoidins from coffee, balsamic vinegar, and sweet wine are the most antioxidant melanoidins consumed in a Spanish diet.^52^ To investigate the relationship between the color intensity and antioxidant properties of the phenol-containing colorants characterized herein, the antioxidant activity of the reaction mixtures was determined by the TEAC assay (Figure 5).

FA/Ara, FA/Ala, and FA/Ara/Ala exhibited an increased color index after 5 min (Figure 1A). At the same time, the antioxidant activity was significantly lower compared to the colorless starting solutions. Subsequent treatment for 10 min resulted in a further decline in the antioxidant properties of all ferulic acid reaction mixtures (Figure 5A), whereas the color index only increased significantly for FA/Ara and FA/Ala (Figure 1A). Even though individual treatment of FA did not result in a significant color formation (Figure 1A), the antioxidant properties significantly decreased compared to unheated ferulic acid (Figure 5A). The decline in antioxidant activity does not correlate with the conversion of FA/Ara and FA/Ala. Both systems exhibited a comparable antioxidant activity after 5 min of heating but had drastically different remaining concentrations of ferulic acid (80 vs 0%). Instead, the data indicate the following trend: higher color intensity coincides with a stronger decrease in the antioxidant properties. This is evident when comparing FA with the binary and ternary mixtures of FA/Ara, FA/Ala, and FA/Ara/Ala, respectively. By HRMS, the increased color intensity of these mixtures may be associated with the decarboxylation of ferulic acid and the subsequent formation of heterogeneous reaction products with Ara, Ala, and/or heterocyclic Maillard intermediates. The data obtained by the TEAC assay also imply that these products exhibit a lower antioxidant activity compared to native ferulic acid. This could be explained by the radical scavenging mechanism of ferulic acid:^58^ in the first step of the reaction with a radical, a hydrogen atom is transferred from ferulic acid to quench the radical, yielding a stabilized phenoxy radical. In the second step, the phenoxy radical can be neutralized by a coupling reaction with another phenoxy radical, yielding a dimer with the capacity to eliminate further radicals. Therefore, we assume that the radical scavenging activity of ferulic acid and *ortho*-methoxy-substituted phenols, in general, is strongly dependent on the steric of these compounds, as the steric hindrance of the larger products (presumably heterogeneous colorants) might strongly influence the coupling rate of two methoxy radicals and thus the regeneration of radical scavenging species. Consequently, the antioxidant properties of *ortho*-methoxy-substituted phenols are hypothesized to decrease with increasing steric hindrance. The observed trend that a higher color intensity results in a decreased antioxidant activity is plausible because both properties are linked by the steric hindrance and the corresponding molecular weight of the colored reaction products. A larger molecular size might result in an enlarged conjugated π-electron system, and, thus, an increased color intensity.

Unlike ferulic acid, the color formation observed for the reaction systems of caffeic acid coincided with an increase of the antioxidant properties (Figure 5B). This may be explained by the heat-induced formation of vinylcatechol oligomers, which exhibit a higher antioxidant capacity compared to native CA.^31^ In contrast to *ortho*-methoxy-substituted phenols, the radical scavenging of vinylcatechol (and its oligomers) does not require a coupling reaction, as both hydroxy groups of each *ortho*-dihydroxybenzene moiety can transfer one hydrogen atom to a radical. Additionally, the conserved hydroxy group stabilizes the radical intermediate through its mesomeric effect, resulting in an increased antioxidant activity of these *ortho*-hydroxy phenols. Consequently, the antioxidant activity of vinylcatechol oligomers might correlate with their degree of oligomerization and is not solely hindered by the steric effect of these molecules. For example, dimeric isomers of vinylcatechol were found to exhibit a two- to three-fold higher antioxidant capacity compared to native CA.^31^ Considering the findings of Plumb et al.,^59^ there is an optimum in the degree of oligomerization and the antioxidant capacity of phenolic compounds with *ortho*-dihydroxybenzene moieties. The authors reported that the antioxidant capacity of epicatechin increased with oligomerization, with the highest radical scavenging activity determined for a trimer, while further oligomerization resulted in a decline of the antioxidant capacity. This concept helps to understand the relationship between color and antioxidant properties of colorants formed in the caffeic acid reaction mixtures: moderate colorants, as formed by incubation of CA and CA/Ara, were presumably stronger antioxidants and smaller oligomers compared to more intense colorants with a lower antioxidant activity which were formed in the reaction mixture of CA/Ara/Ala (Figure 5B). Subsequent heat treatment for 10 min led to an increase in color for CA, CA/Ara, and CA/Ala. However, the antioxidant activity did not change significantly. In the case of CA/Ara/Ala, heat treatment for 10 min resulted in a slight decrease of the antioxidant activity compared to the reaction mixture obtained after 5 min. Consequently, the relationship between the color intensity and the antioxidant activity of the reaction products formed in the mixtures of caffeic acid showed analogies to coffee melanoidins, with moderate colorants exhibiting the highest antioxidant properties. This demonstrates that the incorporation of vinylcatechol into complex, heterogeneous oligomers could indeed explain their different physicochemical properties compared to “classic” melanoidins.

In general, the results were not affected by the type of sugar used in the reaction mixtures. The data obtained for FA/Gal, FA/Gal/Ala, CA/Gal, and CA/Gal/Ala (Figure S-6A,B) were comparable to those of the reaction systems containing Ara.

As to be expected, the pyrolysis of Ara, Ara/Ala, Gal, and Gal/Ala and the heat treatment of pure Ala at 220 °C did not yield soluble antioxidants (Figure S-6C).

### Contribution of Hydroxycinnamic Acids to the Properties of Heterogeneous and Antioxidant Colorants

In their native form, hydroxycinnamic acids exhibit beneficial effects for human health^2,3^ and the stability of food.^4^ However, the chemical modifications induced by thermal processing, like roasting, grilling, or cooking, and their implications on human health are not known to date. The present study provides an in-depth characterization of the complexity of the heat-induced reactions that occur during roasting of phenol-containing food.

In contrast to earlier studies, which primarily discuss that phenolic compounds inhibit the propagation of the Maillard reaction by reducing the concentration of vital intermediates,^13,14,16,17^ we showed that hydroxycinnamic acids play a catalytic role in the formation of heterocyclic Maillard reaction intermediates. Moreover, we demonstrated that ferulic acid and caffeic acid lost their catalytic properties after their heat-induced decarboxylation. Although the corresponding decarboxylation products were no longer acting as catalysators for sugar degradation, they were found to react as donors in electrophilic aromatic substitution reactions with different carbonyl intermediates formed in the presence of sugars and/or amino compounds. To date, such reactions are predominantly considered to trap reactive carbonyl intermediates, but the present findings provide proof that these reactions could act as the key mechanism preceding the formation of phenol-containing caramel colorants and phenol-containing melanoidins.

The different substitution of ferulic acid and caffeic acid significantly impacted their reactivity in nonenzymatic browning reactions. The low browning potential of ferulic acid was found to be strongly increased by its reaction with sugars to phenol-containing caramel-like colorants or amino compounds, yielding melanin-like colorants. However, the presence of an amino compound like alanine was demonstrated to be crucial for the color formation because it strongly catalyzed the decarboxylation of both hydroxycinnamic acids. Besides the increased decarboxylation rate, the reaction between the investigated sugars and alanine also promoted the formation of nitrogen-containing, electron-rich heterocyclic compounds, like pyrrole-2-carbaldehyde. Such compounds can be assumed to act as cross-linking agents that significantly contribute to an enlargement of the conjugated π-electron systems and thus the intense color of phenol-containing melanoidins.

The data presented herein demonstrated that hydroxycinnamic acids promoted the heat-induced degradation of sugars to heterocyclic browning precursors, which was attributed to the acidity of the corresponding phenolic acid. Follow-up studies should be conducted to examine whether hydroxycinnamic acids may also catalyze the degradation of polysaccharides, such as starch, arabinogalactans, or galactomannans. This could accelerate nonenzymatic browning reactions by releasing monomeric sugars that subsequently contribute to browning reactions during processing of plant-based food, such as coffee, cocoa, or nuts. Subsequently, the reactions proposed herein could contribute to understand the conversion of phenolic compounds, carbohydrates, and amino compounds, yielding intense colored and antioxidant phenol-containing melanoidins.

## Supporting Information Available

Additional data regarding the color formation, reaction kinetic, and the antioxidant activity of the galactose reaction systems as well as of the individually treated reactants; and data obtained by the HRMS analysis of the galactose reaction systems and the assignment of the most relevant signals (PDF)

## Abbreviations

Ala: alanine
ABTS: 2,2′-azino-di(3-ethylbenzothiazoline-6-sulfonic acid)
ANOVA: analysis of variance
Ara: arabinose
APCI: atmospheric-pressure chemical ionization
BSTFA: *N*,*O*-bis(trimethylsilyl)trifluoroacetamide
CA: caffeic acid
FA: ferulic acid
FF: furfural
gal: galactose
HCA: hydroxycinnamic acids
HMF: 5-hydroxymethylfurfural
HRMS: high-resolution mass spectrometry
MGO: methylglyoxal
PA: pyrrole-2-carbaldehyde
TEAC: trolox equivalent antioxidant capacity
TMSC: trimethylchlorosilane
