# Supplementary material for: Influence of Hydroxycinnamic Acids on the Maillard Reaction of Arabinose and Galactose beyond Carbonyl-Trapping
Source: J Agric Food Chem. 2024 Jul 5;72(28):15933–47. doi: 10.1021/acs.jafc.4c02959 (PMC11261603; doi:10.1021/acs.jafc.4c02959)
Supplement: Supplementary file 1 — jf4c02959_si_001.pdf [file jf4c02959_si_001.pdf]

The Influence of Hydroxycinnamic Acids on the MAILLARD Reaction of Arabinose and Galactose Beyond Carbonyl-Trapping

Leon Valentin Bork[1,*,†], Nicolas Proksch[1,2,†], Tobias Stobernack[3], Sascha Rohn[1], Clemens Kanzler[1]

[1]Technische Universität Berlin, Institute of Food Technology and Food Chemistry, Department of Food Chemistry and Analysis, Gustav-Meyer-Allee 25, 13355 Berlin, Germany
[2]Leibniz Institute of Vegetable and Ornamental Crops (IGZ) e. V., Plant Quality and Food Security, Theodor-Echtermeyer-Weg 1, 14979 Grossbeeren, Germany
[3]Federal Institute of Risk Assessment, Department of Chemical and Product Safety, Max-Dohrn-Str. 8–10, 10589 Berlin, Germany

*Email: l.bork@tu-berlin.de; Tel.: +49 30 314 72782
†These authors contributed equally to this work.

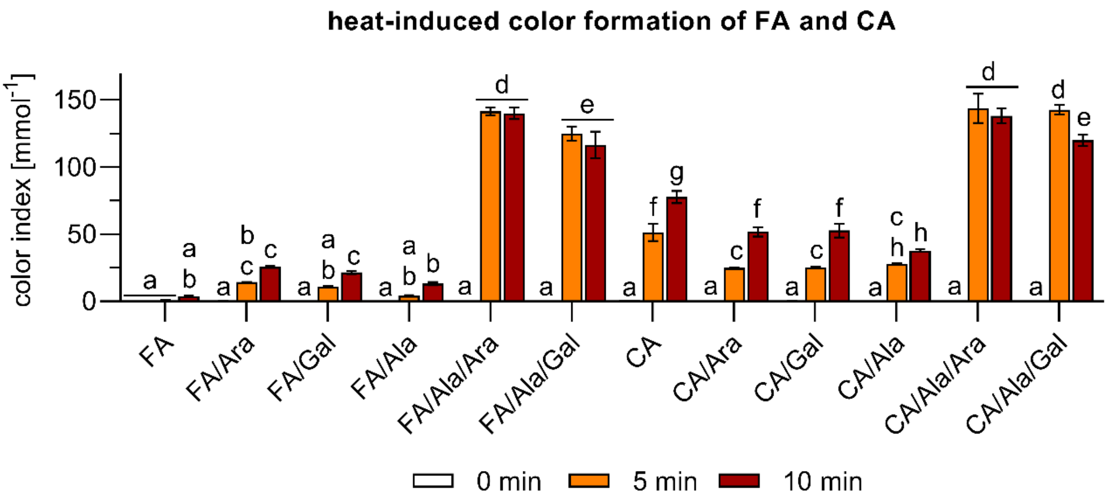

Figure S-1: Comparison of the color formation observed in the different reaction mixture after heat treatment of the individual hydroxycinnamic acids, ferulic acid (FA) and caffeic acid (CA), in binary reaction mixtures with arabinose (Ara), galactose (Gal), or alanine (Ala), as well as in ternary mixtures composed of one hydroxycinnamic acid and sugar with alanine at 220 °C for up to 10 min. Statistical analyses were performed by two-way ANOVA and TUKEY's test ($p < 0.05$). Statistically equal values are designated by equal letters.

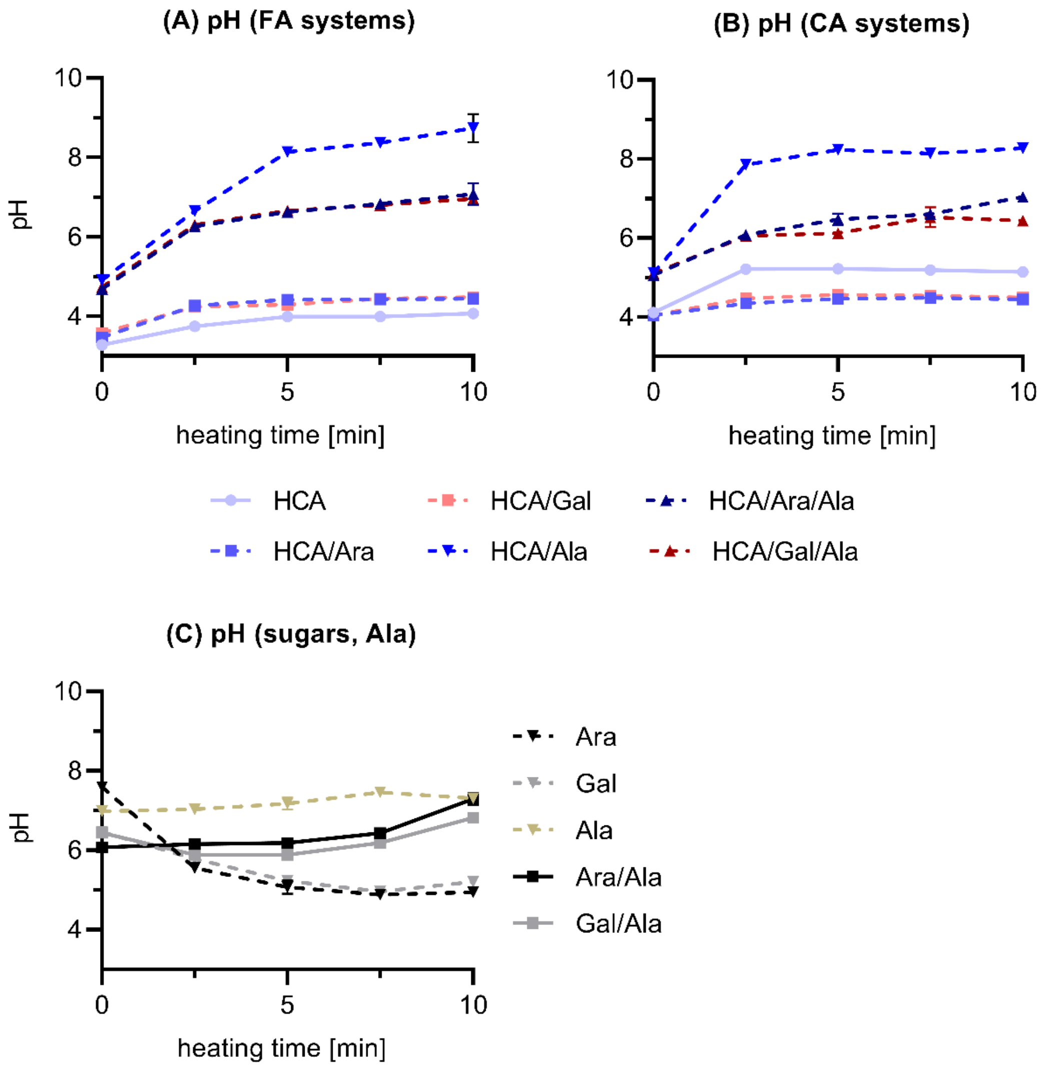

Figure S-2. Heat induced change of the pH value during non-enzymatic browning at 220 °C for up to 10 min of (A) ferulic acid and (B) caffeic acid in combination with arabinose (Ara), alanine (Ala), or galactose (Gal).

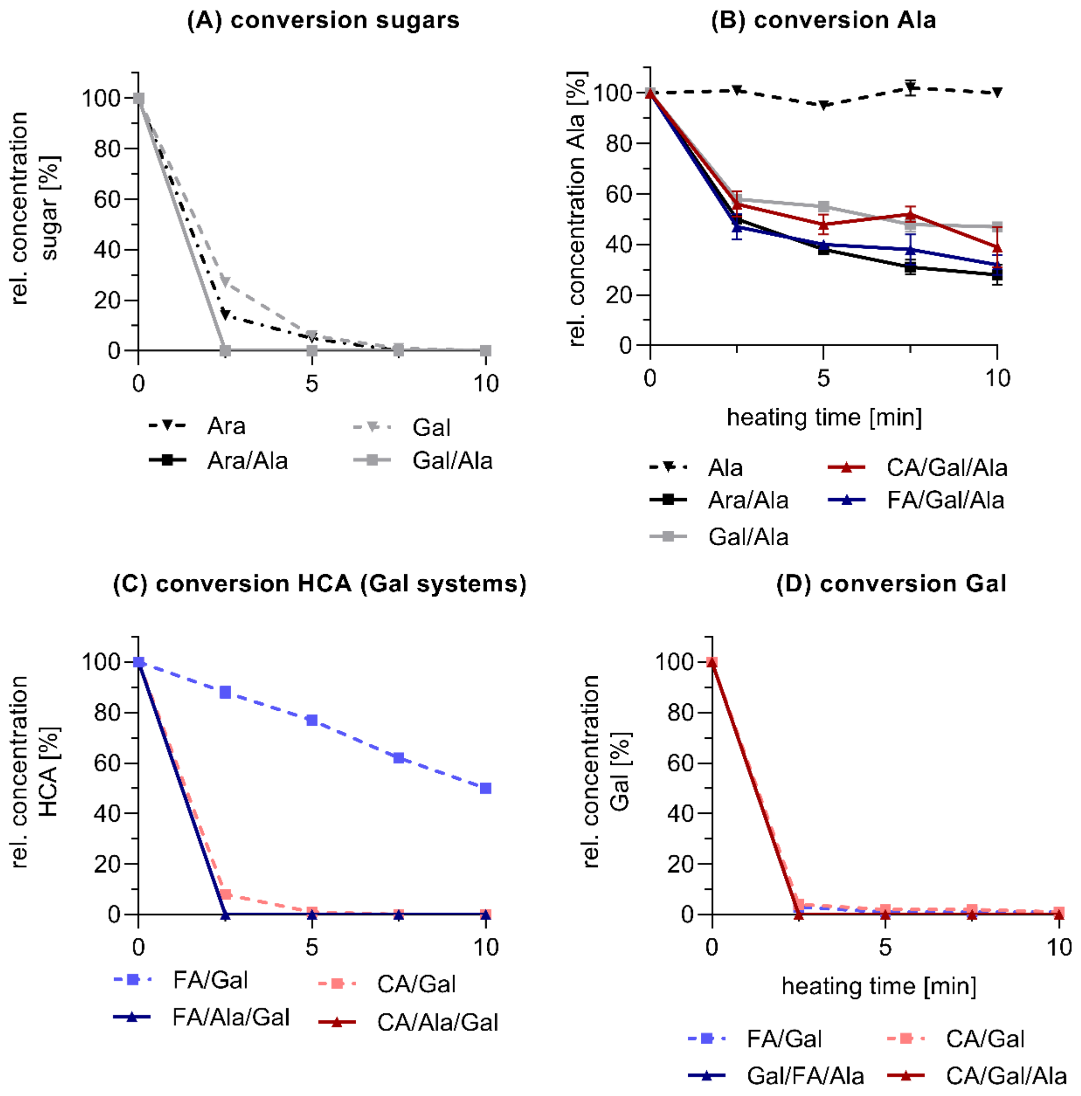

Figure S-3. Heat induced conversion of the hydroxycinnamic acids (HCA) ferulic acid (FA) and caffeic acid (CA) after incubation with galactose (Gal) and/or alanine (Ala). Relative concentration of the sugars (A) Ara and Gal, (B) Ala, (C) the HCA in the Gal systems, and (D) Gal in the corresponding reaction systems after heat treatment at 220 °C for up to 10 min.

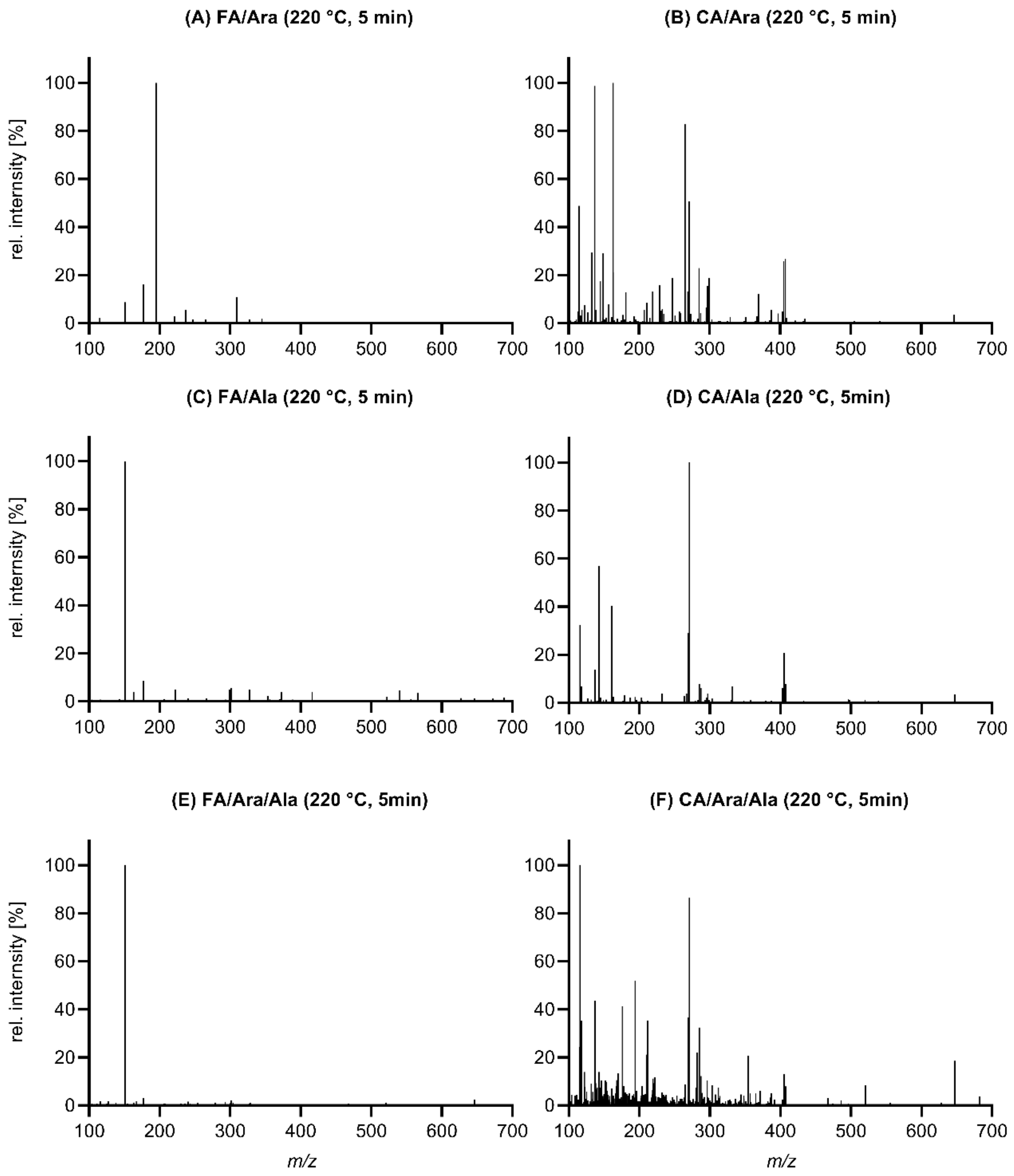

Figure S-4. High-resolution mass spectra obtained after direct injection using atmospheric pressure chemical ionization in positive mode (APCI(+)). Spectra were recorded after heat treatment at 220 °C for 5 min for (A) Ferulic acid/Arabinose (FA/Ara), (B) caffeic acid/arabinose (CA/Ara), (C) ferulic acid/alanine (FA/Ala), (D) caffeic acid/alanine (CA/Ala), (E) FA/Ara/Ala, and (F) CA/Ara/Ala.

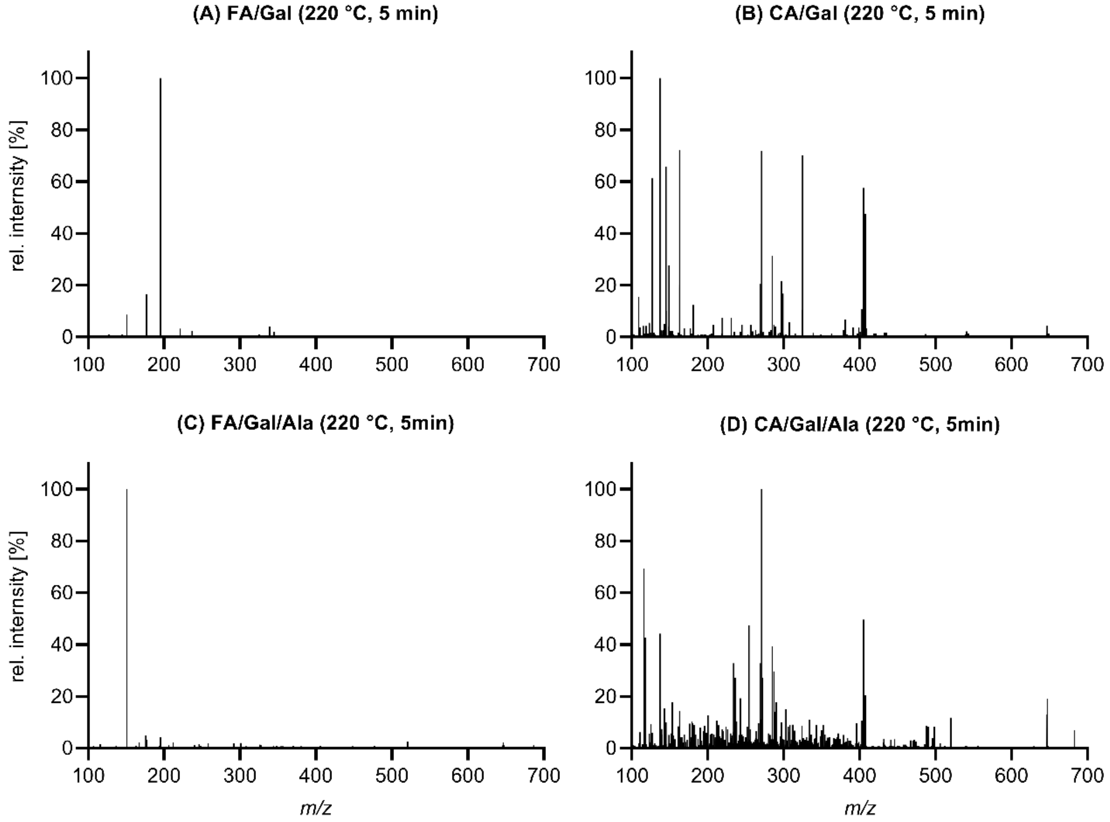

Figure S-5. High-resolution mass spectra obtained after direct injection using atmospheric pressure chemical ionization in positive mode (APCI(+)). Spectra were recorded after heat treatment at 220 °C for 5 min for (A) Ferulic acid/galactose (FA/Gal), (B) caffeic acid/galactose (CA/Gal), (C) ferulic acid/galactose/alanine (FA/Gal/Ala), and (D) caffeic acid/galactose/alanine (CA/Gal/Ala).

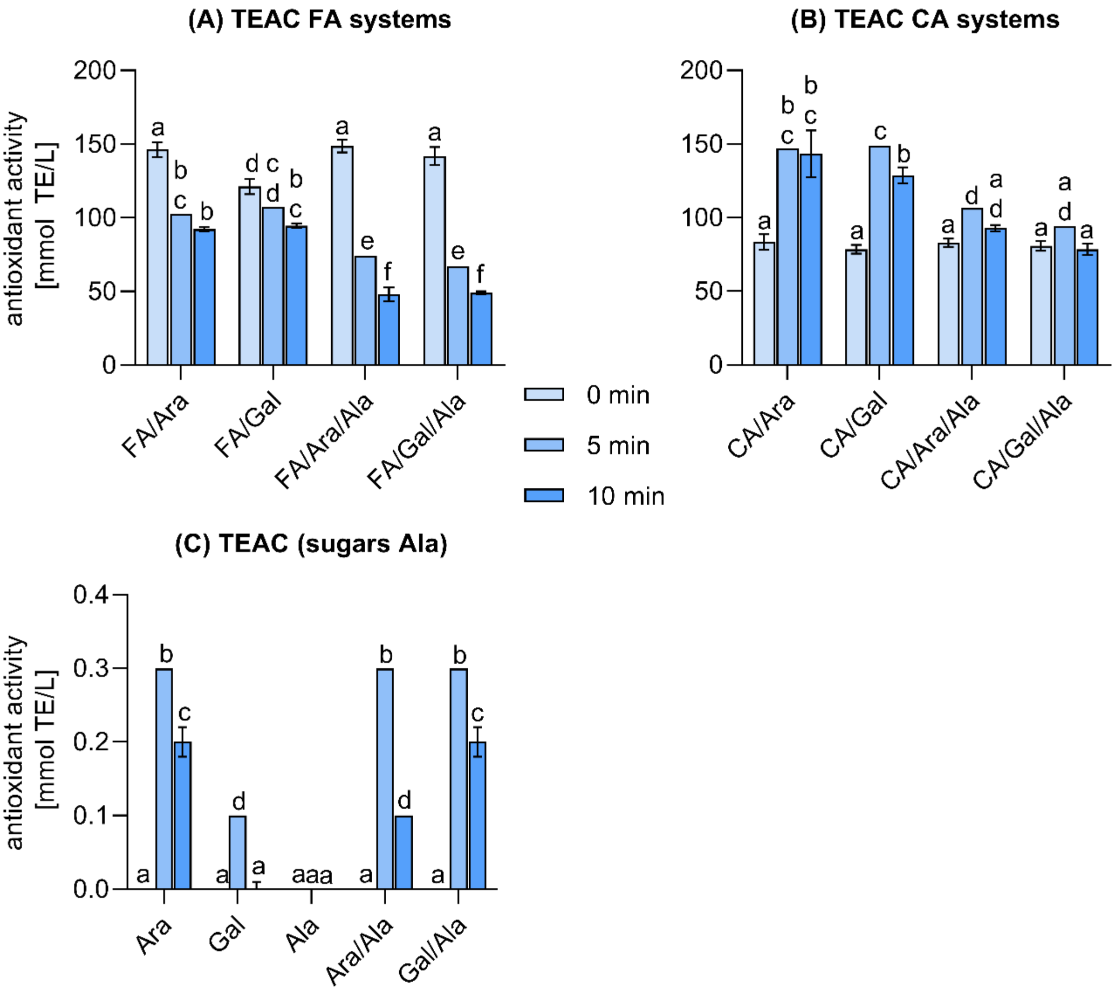

Figure S-6. Antioxidant activity of (A) ferulic acid and (B) caffeic acid incubation with arabinose or galactose as well as the antioxidant activity of (C) the individual sugars, alanine and the corresponding MAILLARD mixtures.

Table S-1. Assignment of selected signals to reaction products detected by APCI(+)-HRMS analysis of the reaction mixtures composed of FA/Gal, CA/Gal, FA/Gal/Ala, and CA/Gal/Ala after heat treatment at 220 °C for 5 min. Only signals with a relative intensity of at least 1 % and a relative error below 5 ppm were considered for the assignment.

reaction mixture	structure assignment						exp. *m/z*	theo. *m/z*	rel. error (ppm)	rel. int. [%]
	compounds			H_2O	H_2	composition				
FA/Gal	1 × HMF	–	–	0	0	$C_6H_6O_2H^+$	127.0389	127.0390	–1.1	1
	1 × Gal	–	–	–2	0	$C_6H_8O_4H^+$	145.0494	145.0495	–0.5	1
	1 × VG	–	–	0	0	$C_9H_{10}O_2H^+$	151.0753	151.0754	–0.5	9
	1 × FA	–	–	–1	0	$C_{10}H_8O_3H^+$	177.0544	177.0546	–1.2	17
	1 × FA	–	–	0	0	$C_{10}H_{10}O_4H^+$	195.0650	195.0652	–1.1	100
	2 × Gal	–	–	–2	0	$C_{12}H_{20}O_{10}H^+$	325.1129	325.1129	–0.0	1
	1 × FA	1 × Gal	–	–2	0	$C_{16}H_{18}O_8H^+$	339.1075	339.1074	0.4	4
	1 × HMF	–	–	0	0	$C_6H_6O_2H^+$	127.0389	127.0390	–1.1	1

	1 × VC	–	–	0	0	$C_8H_8O_2H^+$	137.0594	137.0597	–2.2	100
	1 × VC	1 × HMF	–	–1	–1	$C_{14}H_{10}O_4H^+$	243.0648	243.0652	–1.6	2
	1 × VC	1 × HMF	–	–1	0	$C_{14}H_{12}O_4H^+$	245.0805	245.0808	–1.5	5
	1 × VC	1 × HMF	–	0	0	$C_{14}H_{14}O_5H^+$	263.0910	263.0914	–1.4	3
	2 × VC	–	–	0	0	$C_{16}H_{16}O_4H^+$	273.1117	273.1121	–1.6	2
	1 × VC	1 × Gal	–	0	0	$C_{14}H_{16}O_6H^+$	281.1015	281.1020	–1.5	2
	1 × VC	1 × HMF	1 × MGO	–2	0	$C_{17}H_{14}O_5H^+$	299.0910	299.0914	–1.5	1
CA/Gal	2 × VC	1 × HMF	–	–2	0	$C_{22}H_{18}O_5H^+$	363.1222	363.1227	–1.3	1
	2 × VC	1 × HMF	–	–1	–1	$C_{22}H_{18}O_6H^+$	379.1170	379.1176	–1.6	3
	2 × VC	1 × HMF	–	–1	0	$C_{22}H_{20}O_6H^+$	381.1326	381.1333	–1.7	7
	2 × VC	1 × HMF	–	0	–1	$C_{22}H_{20}O_7H^+$	397.1274	397.1282	–1.9	1
	2 × VC	1 × HMF	–	0	0	$C_{22}H_{22}O_7H^+$	399.1431	399.1438	–1.8	4
	3 × VC	–	–	0	0	$C_{24}H_{24}O_6H^+$	409.1637	409.1646	–2.0	3
	2 × VC	1 × HMF	1 × MGO	–2	0	$C_{25}H_{22}O_7H^+$	435.1429	435.1438	–2.1	2
	3 × VC	1 × Gal	1 × MGO	–2	0	$C_{33}H_{36}O_{12}Na^+$	647.2082	647.2099	–2.6	3
	1 × VG	–	–	0	0	$C_9H_{10}O_2H^+$	151.0753	151.0754	0.5	100
	1 × FA	–	–	0	0	$C_{12}H_{13}O_2NH^+$	195.0651	195.0652	0.5	4
FA/Gal/Ala	1 × VG	1 × Ala	–	0	0	$C_{18}H_{18}O_4H^+$	240.1230	240.1230	0.1	1
	1 × FA	1 × PA	–	0	1	$C_{18}H_{20}O_4H^+$	292.1180	292.1179	–0.5	2
	2 × VG	–	–	0	0	$C_{21}H_{25}O_5NH^+$	301.1435	301.1434	–0.2	2
	1 × VG	1 × PA	1 × FF	–1	1	$C_{30}H_{37}O_8NH^+$	326.1386	326.1387	0.2	1
	1 × VC	–	–	0	0	$C_8H_8O_2H^+$	137.0595	137.0597	–1.6	44
	1 × VC	1 × Ala	–	–2	0	$C_{11}H_{10}O_2NH^+$	190.0860	190.0863	–1.5	8
	1 × VC	1 × Ala	–	0	0	$C_{11}H_{15}O_4NH^+$	226.1071	226.1074	–1.2	7
	1 × VC	1 × FF	–	0	–1	$C_{13}H_{10}O_4H^+$	231.0649	231.0652	–1.0	1
	1 × VC	1 × PA	–	0	0	$C_{13}H_{13}O_3NH^+$	232.0965	232.0968	–1.3	5
	1 × VC	1 × HMF	–	–1	–1	$C_{14}H_{10}O_4H^+$	243.0649	243.0652	–1.3	19
	1 × VC	1 × HMF	–	–1	0	$C_{14}H_{12}O_4H^+$	245.0806	245.0808	–1.0	5
	1 × VC	1 × HMF	–	0	0	$C_{14}H_{14}O_4H^+$	263.0912	263.0914	–0.9	4
	2 × VC	–	–	0	–1	$C_{16}H_{14}O_4H^+$	271.0962	271.0965	–1.2	100
	2 × VC	–	–	0	0	$C_{16}H_{16}O_4H^+$	273.1119	273.1121	–0.8	2
	1 × VC	2 × Ala	–	–2	0	$C_{14}H_{18}O_4N_2H^+$	279.1335	279.1339	–1.5	1
	1 × VC	1 × Gal	–	–2	0	$C_{14}H_{17}O_6H^+$	281.1017	281.1020	–0.9	1
CA/Gal/Ala	1 × VC	1 × Ala	1 × HMF	–3	0	$C_{17}H_{15}O_4NH^+$	298.1072	298.1074	–0.8	2
	1 × VC	2 × Ala	–	0	0	$C_{14}H_{22}O_6N_2H^+$	315.1547	315.1551	–1.2	2
	1 × VC	1 × Ala	1 × HMF	–2	0	$C_{17}H_{17}O_5NH^+$	316.1175	316.1179	–1.3	3
	1 × VC	1 × PA	–	0	0	$C_{16}H_{20}O_5N_2H^+$	321.1441	321.1445	–1.1	3
	1 × VC	1 × Ala	1 × HMF	–1	–1	$C_{17}H_{17}O_6NH^+$	332.1124	332.1129	–1.4	2
	1 × VC	1 × Ala	1 × HMF	0	0	$C_{17}H_{21}O_7NH^+$	352.1386	352.1391	–1.3	9
	1 × VC	1 × PA	1 × HMF	0	0	$C_{19}H_{19}O_6NH^+$	358.1280	358.1285	–1.5	4
	2 × VC	1 × HMF	–	–2	0	$C_{22}H_{18}O_5H^+$	363.1222	363.1227	–1.2	1
	1 × VC	1 × Gal	1 × Ala	–2	0	$C_{17}H_{23}O_8NH^+$	370.1493	370.1496	–0.9	4
	1 × VC	1 × Gal	1 × PA	–2	0	$C_{19}H_{21}O_7NH^+$	376.1386	376.1391	–1.2	2
	2 × VC	1 × HMF	–	–1	–1	$C_{22}H_{18}O_6H^+$	379.1172	379.1176	–1.0	5
	1 × VC	2 × HMF	–	0	0	$C_{20}H_{21}O_8H^+$	389.1227	389.1231	–0.9	2
	2 × VC	1 × HMF	–	0	0	$C_{22}H_{22}O_7H^+$	399.1433	399.1438	–1.3	2
